# Enhancing CRISPR/Cas‐Mediated Gene Knockout With Short Non‐Homologous Oligonucleotides

**DOI:** 10.1111/pbi.70548

**Published:** 2026-02-22

**Authors:** Yen Peng Chew, Aron Ferenczi, Marie Dannay, Cristina Ponce‐Lilly, Adam Kovac, Dávid Tóth, Szilvia Z. Tóth, Attila Molnar

**Affiliations:** ^1^ Institute of Molecular Plant Sciences University of Edinburgh Edinburgh UK; ^2^ Institute of Plant Biology HUN‐REN Biological Research Centre Szeged Hungary

**Keywords:** Chlamydomonas, CRISPR/Cas, gene knockout, KU70/80 heterodimer, non‐homologous oligonucleotide enhancement

## Abstract

*Chlamydomonas reinhardtii* is a model green microalga that has great industrial potential as a sustainable bio‐factory for recombinant protein and high‐value chemical production. Efficient genome editing tools are required to redesign this organism for synthetic biology applications. CRISPR‐Cas editing technologies have already been adapted for gene knockout, transgene knock‐in, and precise gene editing in *C. reinhardtii* . However, the efficacy of CRISPR/Cas‐mediated gene knockout (KO) is low, which hampers pathway engineering and functional genomic studies. Here we report that co‐delivery of CRISPR‐Cas gene editing reagents with short double‐stranded non‐homologous oligodeoxynucleotides (dsNHO) increases gene knockout efficacy up to 100‐fold in *C. reinhardtii* . This phenomenon, referred to as non‐homologous oligonucleotide enhancement (NOE), is heavily affected by the length, structure, and chemical modifications of dsNHO, and is largely mediated by the DNA double‐stranded break sensor KU70/80 (KU) heterodimer in a Cas nuclease‐, locus‐, and strain‐independent manner. Our data suggest that dsNHOs disrupt the cell's double‐stranded break (DSB) sensing pathways, consequently shifting the balance of DNA repair from canonical non‐homologous end joining (c‐NHEJ) towards the more error‐prone, microhomology‐mediated end joining (MMEJ), which could be harnessed as a strategy for improving gene KO efficiency in *Chlamydomonas* and beyond.

## 1 Introduction

The green microalga *Chlamydomonas reinhardtii* has been developed as a model system for genetic and metabolic engineering (Scaife et al. 2015). To generate nuclear gene knockouts (KOs), purified CRISPR‐Cas proteins—such as Cas9 and the ortholog Cas12a (previously known as Cpf1)—along with their cognate guide RNAs (gRNAs), can be delivered as ribonucleoprotein (RNP) complexes into *C. reinhardtii* (Baek et al. 2016; Shin et al. 2016; Dhokane et al. 2020; Ferenczi et al. 2024).

The CRISPR RNPs induce double‐strand DNA breaks (DSBs) in a sequence‐specific manner, which are predominantly repaired by the canonical non‐homologous end‐joining (c‐NHEJ) DNA repair pathway in vegetative cells (Zelensky et al. 2017; Clerici et al. 2008; Falzon et al. 1993). c‐NHEJ is mediated by the KU70/80 (KU) heterodimer complex, which binds DSBs and subsequently recruits other auxiliary factors, such as DNA‐PKcs and DNA ligase IV, to ligate the DNA ends without the use of an additional repair template (Zelensky et al. 2017; Clerici et al. 2008; Falzon et al. 1993). However, this process is error‐prone, especially when repeated DNA cleavage occurs, eventually leading to short insertions and deletions (indels) at the CRISPR target site. In *Chlamydomonas*, the efficacy of CRISPR‐RNP‐induced KOs is relatively low—ranging from 10^−6^ to 5.2% (Baek et al. 2016; Shin et al. 2016; Dhokane et al. 2020; Ferenczi et al. 2024; Jiang and Weeks 2017; Xue et al. 2019) and varies depending on the *C. reinhardtii* strains used, the presence of the cell wall, the type and quality of purified Cas protein, position‐effect of the target site and various other factors (Kim et al. 2020; Camperi et al. 2021).

To increase KO efficacy, CRISPR RNPs can be co‐delivered with double‐stranded DNA (dsDNA) antibiotic‐resistance cassettes into *C. reinhardtii* cells (Shin et al. 2016; Kim et al. 2020; Picariello et al. 2020). This approach enables preselection of transformed cells and subsequent identification of on‐target knock‐in (KI) events of the selection marker at the CRISPR–Cas‐induced DSBs. The addition of homology arms—20–45 nt sequences flanking the Cas cut site—to the antibiotic‐resistance gene can further enhance KI efficiency by exploiting the microhomology‐mediated end joining (MMEJ) DNA repair pathway involving polymerase theta (Pol θ) (Picariello et al. 2020; Ferenczi et al. 2021; Angstenberger et al. 2020; Nievergelt et al. 2023; Sizova et al. 2021). However, the limited number of available selection‐marker genes can constrain the stacking of multiple mutations. It has also been demonstrated that introducing single‐stranded oligonucleotides with 30–45 nt homology arms can increase editing efficiency and enable the precise insertion of STOP codons or short affinity tags at the CRISPR target site in an indefinite series, without the need for additional selection‐marker genes (Xue et al. 2019; Ferenczi et al. 2021, 2017; Greiner et al. 2017; Akella et al. 2021; Ross et al. 2024). However, the size of integrated sequences may be limited due to constraints in the chemical synthesis of single‐stranded oligonucleotides.

**FIGURE 1:**
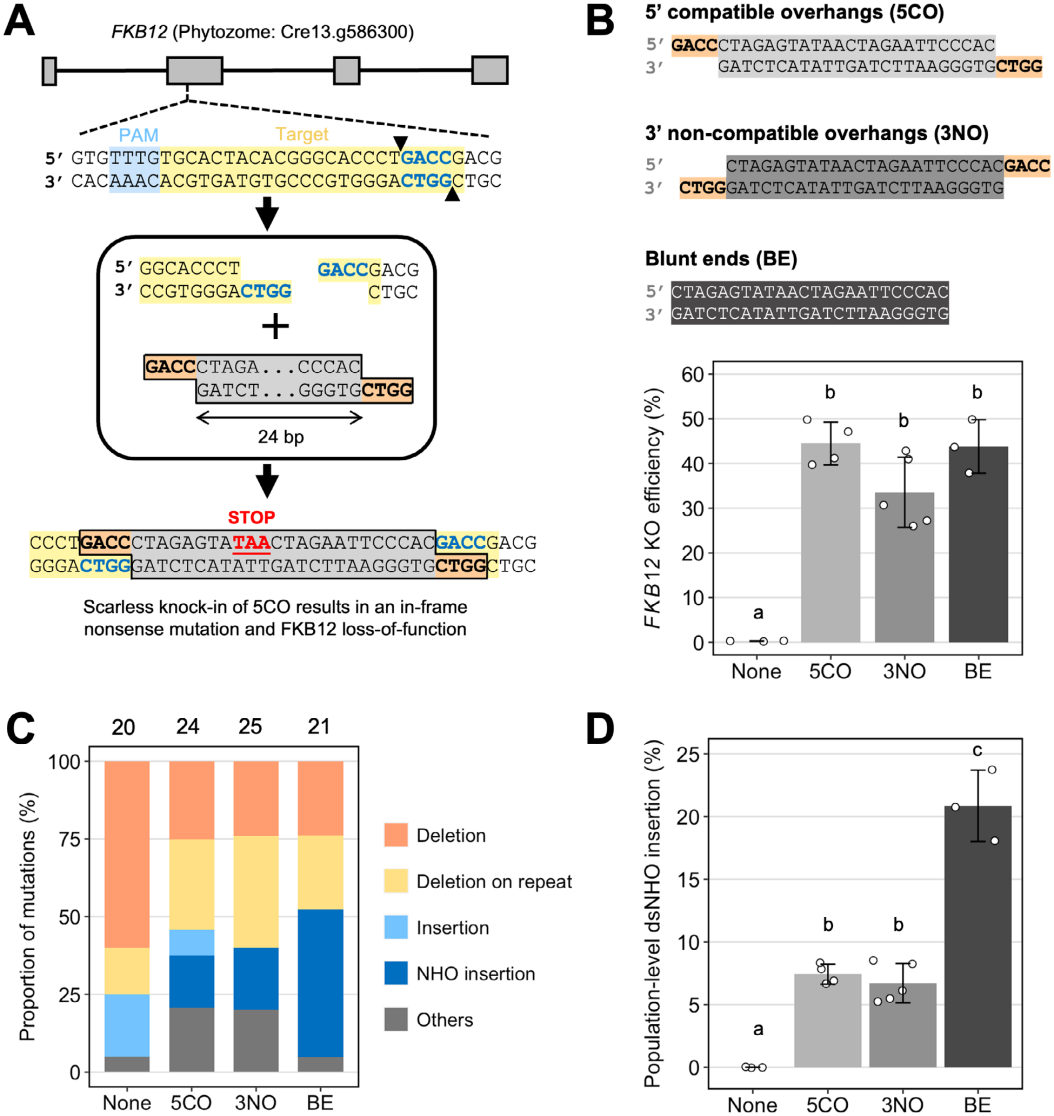
Knock‐in of short exogenous double‐stranded non‐homologous oligodeoxynucleotide (dsNHO) improves FKB12 knockout efficacy in *C. reinhardtii* . (A) Schematic showing the Cas12a TTTV protospacer adjacent motif (PAM) site (light blue), target site (yellow) and expected staggered cleavage (indicated by black triangles) targeting the second exon of the *FKB12* locus. Grey boxes and black lines indicate exons and introns, respectively. We expect a dsNHO with 4‐bp 5′ compatible overhangs (5CO) to be inserted into the Cas12a‐generated 4‐bp overhangs (blue font). A scarless 5CO knock‐in will introduce an in‐frame stop codon into *FKB12*, generating a nonsense mutation and consequent FKB12 functional knockout. (B) The impact of 24‐bp dsNHO termini on the *FKB12* KO efficiency when co‐delivered with Cas12a RNPs (3 ≤ *n* ≤ 5). Bars labelled with different letters were significantly different (*p* < 0.01) according to one‐way ANOVA test. The error bars represent the standard deviation. (C) Analysis of editing events at the *FKB12* cut site of colonies treated with Cas12a alone, or with 5CO, 3NO and BE dsNHO. The number of successful colony PCR and Sanger sequences analysed are indicated at the top of the bars. The ‘Deletion on repeat’ category represents sequences that potentially have microhomology‐mediated deletions whereas ‘Deletion’ indicates deletions that do not. ‘NHO insertion’ represents any sequences that have homology to dsNHO and ‘Insertion’ represents sequences that do not have homology. The ‘Others’ category is a mixture of mismatches and unknown sequences that end abruptly in the Sanger chromatogram. (D) The population‐level dsNHO insertion was calculated by multiplying the average *FKB12* KO efficiency with the ratio of ‘NHO insertions’ category.

Cas9 generates blunt‐ended DSBs three nucleotides upstream of the protospacer‐adjacent motif (PAM), whereas Cas12a induces a PAM‐distal, staggered DSBs with a 4‐ or 5‐nt 5′ overhang located 17–23 nt downstream of the PAM (Zetsche et al. 2015). The DNA ends at the Cas12a cut site resemble the overhangs generated by many type II restriction endonucleases. Therefore, we sought to investigate the precision knock‐in of double‐stranded non‐homologous oligodeoxynucleotides (dsNHO) with complementary sticky ends via homology‐mediated ligation into the Cas12a‐generated staggered DSBs (Figure 1A).

We report that the addition of short sticky ends to exogenous dsNHOs was insufficient to allow precise knock‐in into the Cas12a‐induced staggered DSBs. However, the co‐delivery of short exogenous dsNHOs was found to boost KO efficiency by up to 100‐fold in a locus‐, Cas nuclease‐, and alga strain‐independent manner. We show that this phenomenon, termed non‐homologous oligonucleotide enhancement (NOE), is primarily mediated by the KU heterodimer involved in the c‐NHEJ DSB repair pathway, rather than by Pol θ involved in MMEJ (Xue et al. 2019; Sizova et al. 2021; Black et al. 2016; Chang et al. 2017).

## 2 Results

To estimate CRISPR‐mediated gene KO efficacy in *C. reinhardtii* (cc‐1883 *cw15*), we targeted the *FKB12* locus (Cre13.g586300), where loss‐of‐function leads to rapamycin resistance (Ferenczi et al. 2017). We delivered *FKB12*‐targeting Cas RNPs and other agents via electroporation into cells and quantified *FKB12* KO efficiency as the proportion of viable cells able to grow on solid plates supplemented with rapamycin.

We first examined the insertion of a short dsNHO with 5′ sticky ends into a Cas12a‐generated 4‐bp staggered overhang (Figure 1A). We designed a 24‐bp dsNHO that had no homology to *FKB12*, except in its 5′ sticky ends (5CO). Scarless insertion of 5CO would produce a truncated, non‐functional FKB12 protein due to the introduction of an in‐frame stop codon (Figure 1A).

When Cas12a RNPs targeting *FKB12* were delivered on their own, we obtained a low average *FKB12* KO efficiency of 0.29% (Figure 1B). However, when 1 mM of 5CO was co‐delivered with Cas12a RNPs, KO efficiency increased approximately 150‐fold to 44.5% (Figure 1B). Notably, no rapamycin‐resistant colonies were recovered using 5CO alone (Figure S1A). Next, we amplified and sequenced the genomic region encompassing the Cas12a cut site from a set of randomly selected rapamycin‐resistant colonies to understand how 5CO affects DNA repair at the Cas12a‐induced DSB. We observed a mixture of random and microhomology‐mediated deletions (i.e., deletion on repeat), and dsNHO insertions in the cut site (Figure 1C and Figure S1B, C). However, none of the knock‐ins of 5CO were scarless (Figure 1C, D and Figure S1C). Instead, we observed scarred knock‐in of 5CO, concatenations of 5CO, as well as inverted insertion of 5CO into the DSB (Figure S1C). One explanation is that the sticky ends of 5CO had too little homology to allow precise knock‐in to the DSB. However, it appeared that 5CO could enhance overall KO efficiency.

To understand whether the KO enhancement was due to the dsNHO termini, we designed two additional dsNHOs; one with 3′ overhangs that were not complementary to the Cas12a‐generated DSB (3NO) and one with blunt ends (BE). Both dsNHOs also enhanced *FKB12* KO efficiency as well as 5CO (5CO = 44.5%, BE = 43.8%, 3NO = 33.6%, *p* < 0.001; Figure 1B). The BE dsNHO was inserted into the expected DSB significantly more often than 5CO and 3NO on a population‐level (BE = 20.9%, 5CO = 7.41%, *p* < 0.001; 3NO = 6.72%, *p* < 0.001; Figure 1C,D and Figure S1D, E), suggesting that the DNA repair mechanism in *C. reinhardtii* prefers to integrate blunt dsNHOs, despite the staggered DSB generated by Cas12a. We next tested the effect of the internal part of the dsNHO sequence, using dsNHOs with 3′ overhangs consisting of four random nucleotides, to remove any effect of the sequence of the overhangs (Figure S2A). Different sequences with the same GC content had similar effects (Figure S2B). However, sequences with lower GC content had a greater effect in enhancing KO efficiency, despite annealing to the same extent in vitro (Figure S2C). Importantly, we also found that this enhancement is dependent on the concentration of dsNHO, reaching a plateau at around 50% KO efficiency (Figure S3).

The enhancement in KO efficiency by use of dsNHO alongside Cas12a that we observed in *C. reinhardtii* had also been seen in mammalian cells using Cas9 (Richardson et al. 2016); though with only a moderate 2–3‐fold increase in gene knockout efficacy. This phenomenon, presumably due to NHOs shifting cells towards error‐prone repair, has been dubbed non‐homologous oligonucleotide enhancement (NOE), a term which we now adopt here.

**FIGURE 2:**
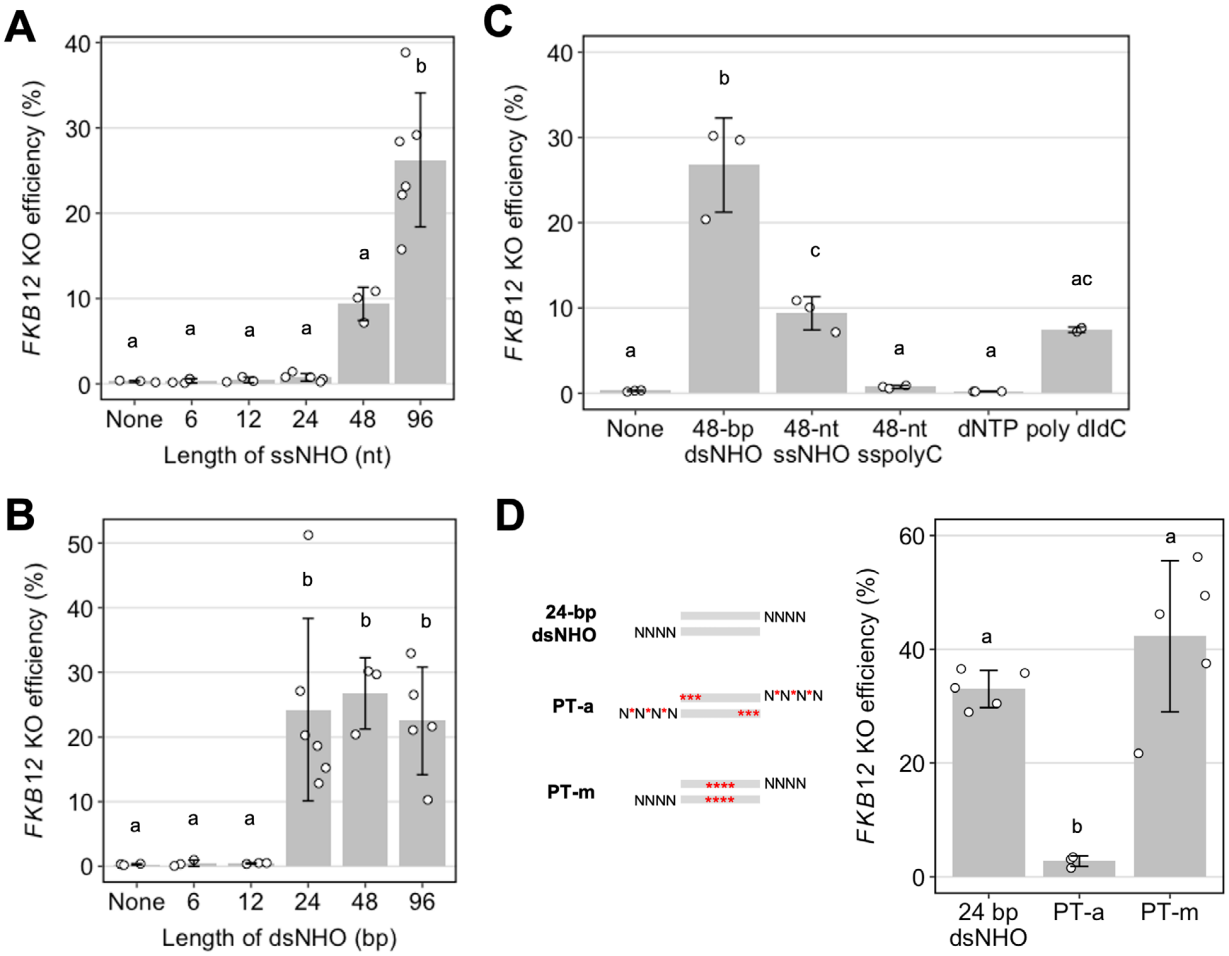
The impact of NHO length and strand type on the *FKB12* KO efficacy in *C. reinhardtii* . *FKB12* KO efficiency of cells treated with Cas12a RNPs alone or with different lengths of (A) single‐stranded, or (B) double‐stranded NHO with NNNN overhangs (3 ≤ *n* ≤ 6), (C) 48‐nt cytosine homopolymer (polyC), deoxynucleotides (dNTPs), poly deoxyinosinic‐deoxycytidylic acid (poly dI‐dC) or (D) 24‐bp dsNHOs with PT modifications at the termini (PS‐a) or in the middle of the dsNHO sequence (PS‐m). Bars labelled with different letters were significantly different (*p* < 0.05) according to one‐way ANOVA test. The error bars represent the standard deviation.

We next investigated the optimal length of NHO by delivering different lengths of single‐stranded NHO (ssNHO) or dsNHO. Co‐delivery of short (6–24 nt) ssNHO (forward strand of relevant dsNHO used) did not significantly increase *FKB12* KO efficiency compared to the use of Cas12a alone, whereas long ssNHOs such as 96 nt did (none = 0.30%, 96 nt = 26.3%, *p* = < 0.001, Figure 2A). For dsNHOs, in contrast, a 24 bp length was sufficient to induce NOE (none = 0.30%, 24 bp = 24.2%, *p* = < 0.001) and efficiency did not increase further with longer dsNHOs (Figure 2B). Rapamycin‐resistant colonies showed a mixture of insertions and deletions at the Cas12a target site, and there was no correlation to the proportion of mutation types with the length of NHOs used (Figure S4A, B).

The difference in the effect of NHO size between ssNHOs and dsNHOs led us to hypothesise that NOE is dependent on double‐stranded DNA (dsDNA), because longer ssNHOs have greater potential to self‐anneal to form double‐stranded regions than shorter ones (Figure S4C). To test this, we co‐delivered 48‐nt cytosine homopolymer (polyC), which has no capacity for self‐annealing, with *FKB12* Cas12a RNP. We observed that it did not increase *FKB12* KO to the same extent as 48‐nt ssNHO or 48‐bp dsNHO (polyC = 0.77%, 48‐nt ssNHO = 9.40%, *p* = 0.015; 48‐bp dsNHO = 26.8%, *p* < 0.001; Figure 2C). Similarly, a 24 nt ssNHO designed to have no ability to form strong secondary structure caused no detectable KO enhancement (none = 0.30%, 24‐nt ssNHO = 0.89%, *p* = 1.000, Figure S4 and Figure 2A).

Our hypothesis was further supported by the absence of NOE upon co‐delivery of deoxynucleotides (dNTPs) or a dsDNA analogue (1200–3000 bp long) comprising a co‐polymer of deoxyinosinic‐deoxycytidylic acid (poly dI‐dC) (none = 0.03%, dNTPs = 0.21%, *p* = 1.000; poly dI‐dC = 7.46%, *p* = 1.000; Figure 2C). Additionally, we tested whether RNA of different lengths or secondary structures can stimulate NOE and found no significant increase in *FKB12* KO efficiency when it was co‐delivered with Cas12a (Figure S5B), which contrasts with observations in human cells using Cas9 RNPs (Richardson et al. 2016).

We next explored the potential to further increase *FKB12* KO efficiency using dsNHO with chemical modifications, such as phosphorothioate bonds (PT) and locked nucleic acids (LNAs), that are known to increase intracellular retention of double‐stranded oligonucleotides and slow down degradation by exonucleases (Thierry and Dritschilo 1992; Hosoya et al. 1999; Crinelli et al. 2002; Figure S6). Addition of PT modifications to both 5′ and 3′ of the dsNHO (PT‐a) almost abolished NOE (unmodified = 33.01%, PT‐a = 2.76%, *p* = 0.002, Figure 2D). In contrast, PT modifications in the middle of the dsNHO (PT‐m) slightly enhanced FKB12 KO efficiency (unmodified = 33.01%, PT‐m = 42.49%, *p* = 0.255, Figure 2D).

These observations suggested that the accessibility of the DNA overhangs may be important, perhaps indicating the involvement of DNA termini‐binding proteins. We further explored this idea by annealing multiple combinations of dsNHO with various PT and LNA modifications (Figure S6B). The results showed that PT modification of both 5′ ends of the dsNHO was sufficient to reduce its effect on NOE.

Our findings led us to speculate that the exogenous dsNHOs function as decoys by diverting DNA repair proteins from the Cas12a cut site, leading to more error‐prone repair and increased KO efficiency (Figure S7). Candidate DNA repair proteins of interest include the KU heterodimer, which binds DSBs and initiates the c‐NHEJ pathway (Clerici et al. 2008; Falzon et al. 1993). The minimum length of dsDNA required for KU binding is generally considered to be around 14–18 bp (Falzon et al. 1993; Yaneva et al. 1997; Walker et al. 2001), which could explain why 24‐nt dsNHOs were sufficient, whereas 6‐ and 12‐bp dsNHOs were inefficient in NOE (Figure 2B). Intriguingly, the KU heterodimer can also interact with hairpin DNA (Clerici et al. 2008), which corroborates our finding that longer ssNHOs with the capacity to form dsDNA can enhance KO efficiency (Figure S4). Another candidate of interest is the MRN (MRE11‐RAD50‐NBS1) complex (Syed and Tainer 2018) involved in DSB end resection and activation of the ATM pathway (Clerici et al. 2008; Falzon et al. 1993; Chang et al. 2017). Because we had observed an increase in microhomology‐mediated deletions when NHOs were co‐delivered with Cas12a RNPs, we also investigated the involvement of Pol θ—encoded by the *POLQ* gene—which is a key player in MMEJ repair of DSBs in *C. reinhardtii* (Ferenczi et al. 2021; Sizova et al. 2021; Black et al. 2016).

We generated *ku70, ku80, mre11*, and *polq* mutants by knock‐in (KI) of the *aphVIII* cassette conferring paromomycin resistance, using different Cas proteins (Figure S8). Adding 50‐bp homology arms to *aphVIII* almost doubled on‐target KI efficiency in asynchronous (blunt = 12.5%, homology arms = 29.2%) and synchronous cells (blunt = 37.5%, homology arms = 66.7%, Figure S8), supporting previous results (Picariello et al. 2020; Angstenberger et al. 2020). The antibiotic resistance gene KI was chosen to maintain consistency with the technology employed to generate mutants in DSB repair pathways in earlier studies, including those targeting *Ku70* and *PolQ*. At least three independent mutants for each targeted gene were selected based on unique gene‐*aphVIII* junction sequence (Figure S9; Ferenczi et al. 2021), with the exception of the *kupolq* double knockout, where only two strains were available. We examined the effects of the mutations on DNA repair by testing sensitivity to zeocin and camptothecin, which cause both DSB and single‐stranded breaks (Figure S10). The *mre11, polq1*, and *kupolq* double mutants were hypersensitive to both agents, whereas *ku* mutants showed only slight sensitivity to them, suggesting that MRE11 and Pol θ may have additional roles in cell function such as DNA replication during stress (Syed and Tainer 2018; Kelso et al. 2019).

**FIGURE 3:**
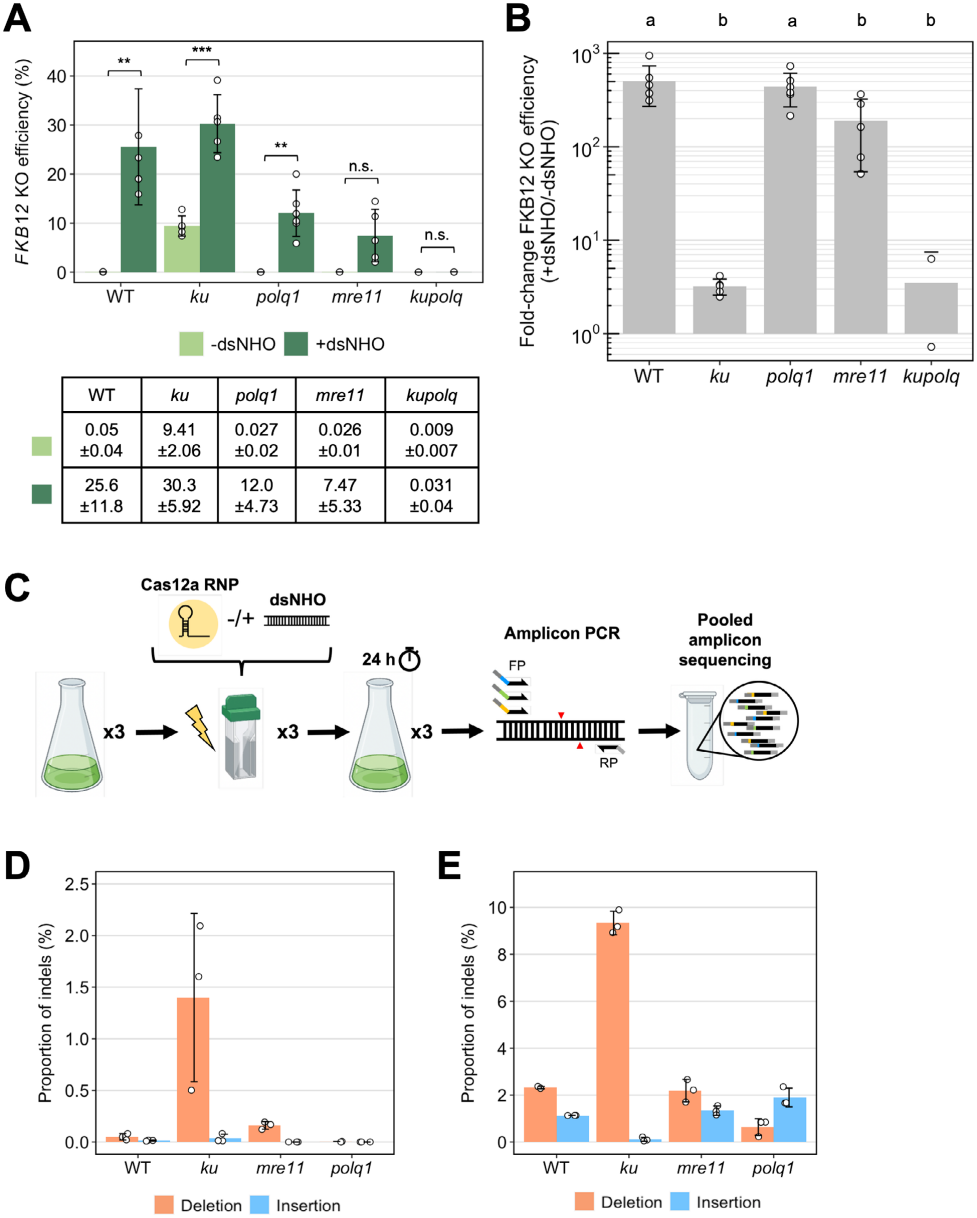
Investigating NOE in *C. reinhardtii* DNA repair mutants. (A) Asynchronous wild‐type (*n* = 3), *ku* (*n* = 3), *polq1* (*n* = 3), *mre11* (*n* = 3), *kupolq* double mutant (*n* = 2) were transformed with *FKB12* Cas12a RNP with and without 24‐bp dsNHO. Average *FKB12* KO efficiencies and standard deviation are stated in the table below. One‐way ANOVA test significance values are represented by asterisks. *p < 0.05, **p < 0.01, ***p < 0.001, n.s.: not significant. The error bars represent the standard deviation. (B) Fold‐change *FKB12* KO efficiency between Cas12a RNP delivery and co‐delivery with dsNHO. Bars labelled with different letters were significantly different (*p* < 0.05) according to one‐way ANOVA test. (C) Diagram showing methodology used for pooled amplicon PCR to analyse editing events in a cell population. Percentage of edited reads with insertion or deletion mutations at the *FKB12* target site in strains transformed with *FKB12* Cas12a RNP without (D) and with (E) 24‐bp dsNHO. The mutations are analysed by CRISPResso2 (Clement et al. 2019).

We then used the *fkb12* assay to determine whether Cas12a‐mediated KO efficiency is reduced in DNA repair mutants compared to wild‐type (WT). When Cas12a RNP was delivered alone, we observed low *FKB12* KO efficiencies in WT, *polq1*, and *mre11* strains of between 0.025% and 0.050% (Figure 3A). In contrast, KO efficiency was around 200 times higher (9.41%) in *ku* mutants compared to WT (*p* < 0.001, Figure 3A). This strongly suggested that c‐NHEJ is the main pathway involved in Cas12a‐mediated DSB repair and error‐prone MMEJ‐mediated repair occurs in its absence. In *kupolq* double mutants, the increase in KO efficiency was abolished (WT = 0.05%, *kupolq* = 0.01%, *p* = 1.000, Figure 3A), supporting the view that the c‐NHEJ and MMEJ pathways work in a hierarchical manner to repair Cas12a‐mediated DSBs.

When dsNHO was co‐delivered with Cas12a RNP, we observed a significant increase in KO efficiency in all strains compared to their Cas12a‐only treatment (Figure 3A). To enable comparison between strains, we calculated fold‐change of *FKB12* KO efficiency compared to their respective Cas12a‐only treatments (Figure 3B). We observed 200‐ to 500‐fold changes in KO efficiency in WT and in *polq1* and *mre11* mutants (Figure 3B). The slightly lower fold change in *mre11* suggests a minor role for the MRN complex in NOE. However, the *mre11* lines exhibit a slower growth rate compared to the WT, which could have affected their recovery after electroporation and consequently reduced their editing efficiency (Figure S9B). Importantly, in *ku* and *kupolq* mutants, we saw a significant reduction in fold‐change KO enhancement relative to WT (WT = 502, *ku* = 3.22, *kupolq* = 3.52, Figure 3B). This strongly suggests that the KU heterodimer is required for NOE. Notably, we also confirmed the loss of NOE in *ku* when a different dsNHO sequence was co‐delivered with Cas12a (Figure S11).

To compare the outcomes of DNA repair, we harvested genomic DNA from populations of cells that had been allowed to recover overnight and analysed sequences of pooled amplicons with CRISPresso2 (Figure 3C; Clement et al. 2019). In Cas12a‐only transfections, we observed a significant increase in deletions in *ku* mutants compared to WT (WT = 0.05%, *ku* = 1.4%, *p* < 0.001, Figure 3D), consistent with the idea that loss of KU allows error‐prone MMEJ repair to occur (Sizova et al. 2021; Rodgers and McVey 2016). We also detected a slight, though not significant, increase in deletion rates in *mre11* mutants to 0.16% compared to WT. In contrast, *polq1* mutants had a roughly 25‐fold lower frequency of deletions compared to WT (WT = 0.05%, *polq1* = 0.002%, *p* = 0.003), supporting the view that deletions are largely produced by the MMEJ pathway (Sizova et al. 2021; Black et al. 2016; Kelso et al. 2019).

When dsNHO was co‐delivered with Cas12a RNP, we saw an increase in the combined insertions and deletions in all strains compared to use of the RNP alone (compare Figure 3D,E), consistent with the increased frequency of *FKB12* KOs. However, the frequency of insertions in the *ku* mutants significantly decreased, indicating that KU heterodimer is a major factor responsible for dsNHO insertions into DSBs in *C. reinhardtii* . This is supported by the higher insertion frequencies compared to deletions in *polq1* mutants where c‐NHEJ is suspected to be the major DSB repair pathway (Figure 3D).

**FIGURE 4:**
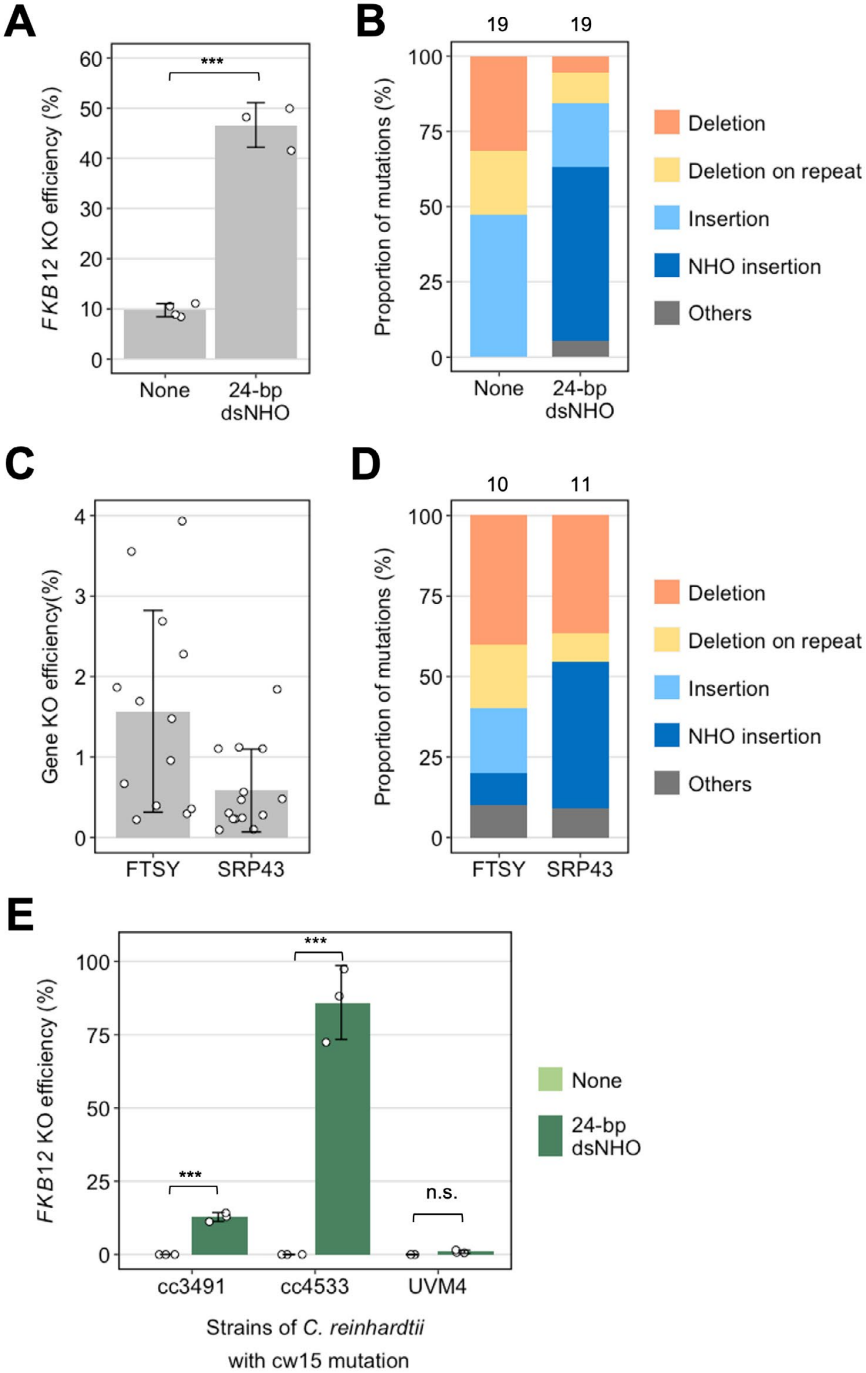
Investigating NOE in other *C. reinhardtii* strains and locus. (A) *FKB12* KO efficiency of cells transformed with Cas9 RNP without or with 24‐bp dsNHO and (B) the analysis of editing events at the respective cut site of rapamycin‐resistant mutants. Bars labelled with different letters were significantly different (*p* < 0.05) according to one‐way ANOVA test. (C) Gene knockout efficiency of wild‐type cells that were transformed with *FTSY* or *SRP43* Cas12a RNP with 24‐bp dsNHO and (D) the analysis of editing events at the respective cut site of light‐green mutants. (E) *FKB12* KO efficiency in different *C. reinhardtii* strains with cw15 mutation with and without co‐delivery of 24‐bp dsNHO alongside Cas9 RNP. One way ANOVA test significance values are represented by asterisks. *p < 0.05, **p < 0.01, ***p < 0.001, n.s.: not significant. The error bars represent the standard deviation.

We then examined whether Cas9 RNPs had a similar effect to Cas12a. Importantly, NOE was also observed using Cas9 (none = 9.8%, dsNHO = 46.6%, *p* ≤ 0.001, Figure 4A). However, more than half of the KOs involved concatenated dsNHO insertions into the DSB in the case of Cas9 (Figure 4B and Figure S12), compared to only around 15% with Cas12a. This suggests that differences in the kinetics of Cas12a and Cas9 binding or cleavage product release can affect DNA repair outcomes at the DSB (Strohkendl et al. 2018). Further supporting this, we observed small indels of 1–2 bp with Cas9 that were not observed when using Cas12a RNPs (Figures S1 and S12) and were presumably generated by the *C. reinhardtii* c‐NHEJ pathway (Sizova et al. 2021). Instead, Cas12a produced 4–15 bp MMEJ‐mediated deletions (Figure S1). This suggests that the Cas12a‐mediated staggered DSB could promote end resection, consequently increasing the frequency of MMEJ repair over c‐NHEJ. Nevertheless, the addition of dsNHOs increases error‐prone repair at the targeted DSB regardless of the Cas protein used.

To investigate whether NOE is observed at other *C. reinhardtii* loci, we targeted *FTSY* (*CpFTSY*; Cre05.g241450) and *SRP43* (*CpSRP43*; Cre04.g231026), which encode chloroplast‐located proteins. Loss‐of‐function mutants develop a light‐green phenotype and a reduced chlorophyll content that can be observed using blue light (Kirst et al. 2012). With *FTSY* and *SRP43* Cas12a RNP delivery alone, we did not obtain any light‐green colonies after screening over 2300 and 2800 colonies respectively, indicating that the KO efficiency is low (< 0.04%). Co‐delivery of dsNHOs allowed us to detect *ftsy* and *srp43* mutants at an average frequency of 1.57% and 0.58%, respectively (Figure 4C), with a mixture of indels and dsNHO insertions in the light‐green mutants (Figure 4D). This significant increase in gene KO efficacy using dsNHOs suggests that NOE occurred at the *CpFTSY* and *CpSRP43* loci, and that dsNHOs can rescue otherwise ineffective CRISPR reagents. Next, we examined the impact of NOE on KO efficiency where a loss‐of‐function mutation in the target gene is not associated with a strong and selectable phenotype. We targeted *CrPHT4‐7* (Cre16.g663600), an inorganic phosphate (Pi) transporter required for maintaining Pi homeostasis and optimal photosynthesis under high light conditions (Tóth et al. 2024). Cas12a RNP transfection alone resulted in a 2% editing efficiency, as measured by ICE sequence analysis (Conant et al. 2022) of a PCR product spanning the Cas12a target site that had been amplified from a population of recovered cells (Figure S13). In a parallel experiment, Cas12a RNPs were co‐transfected with dsNHO, and the recovered cells were plated on TAP medium. Sequence analysis of 10 randomly selected colonies identified 3 lines with deletions around the Cas12a target site in the *PHT4‐*7 locus (Figure S14A), corresponding to a 30% KO efficiency. Detailed physiological analyses revealed that the KO mutants generated by dsNHO co‐delivery phenocopied the *pht4‐7* mutant lines (Figure S14B–D) that had been previously generated using a different gene‐editing method, single‐strand templated repair (SSTR; Tóth et al.  2024). From the above experiments, we conclude that dsNHOs can significantly enhance KO efficiency in different loci, and the resulting mutants recapitulate the phenotype of existing CRISPR‐generated lines.

NOE was also observed in three other cell‐wall less *C. reinhardtii* strains, cc‐3491, cc‐4533 and UVM4 where co‐delivery of dsNHO with Cas12a RNP increased FKB12 KO efficiency significantly (Figure 4E), indicating that NOE can be used to enhance gene KO efficiency in other *C. reinhardtii* strains efficiently. However, we noticed that the boost in *FKB12* KO efficiency by dsNHO varies between strains, suggesting that different strains have different ‘sensitivity’ to exogenous DNA, possibly due to different strain‐specific efficiencies of DNA repair pathways.

## 3 Discussion

Our results reveal that dsNHOs at least 24 bp in length can boost gene KO efficacy by up to two orders of magnitude when co‐delivered with Cas12a or Cas9 RNPs, and that ssNHOs capable of forming DNA secondary structures can also produce this effect. In human cells, by contrast, ssNHOs have been reported to outperform dsNHOs, resulting in approximately 3‐ and 2‐fold increases in indel formation at the CRISPR‐Cas9 cut site, respectively (Richardson et al. 2016). These clear qualitative and quantitative differences in NOE between the alga and human cell lines may be attributed to structural and/or functional differences in genes involved in DSB repair; however, further studies will be required to address these questions in both organisms. Importantly, the 127 nt ssNHOs used in human cells were able to form complex secondary structures (Figure S15), supporting our observations that any strong DNA secondary structures can induce NOE (Figure 2C). We have also shown that large DNA copolymers, specifically poly dI‐dC (1.2–3 kb), can also be used to stimulate NOE, albeit at a lower efficiency compared to short dsNHOs due to the molarity difference in DNA ends. Our results also suggest that the type of the dsNHO termini has no significant effect on *FKB12* KO efficacy, but that the termini influence the mutation outcomes around the DSB (Figure 1B–D).

Additionally, we showed that the accessibility of the dsNHO termini is important in stimulating NOE (Figure 2D and Figure S6), suggesting that DNA‐binding proteins may be involved. The titratability of NOE further supports this idea (Figure S3). Thus, we explored the hypothesis of a ‘decoy’ model, in which dsNHOs sequester DNA repair proteins away from the CRISPR‐Cas‐induced DSB, thereby shifting DNA repair from error‐free to error‐prone pathway(s), and consequently increasing the frequency of indels at the CRISPR cut site (Figure S7). Our results strongly suggest that the KU heterodimer is a major player in NOE (Figure 3). Since KU requires a double‐stranded DNA length of approximately 14–18 bp for efficient binding (Falzon et al. 1993; Yaneva et al. 1997; Walker et al. 2001), this may explain why dsNHOs shorter than 24 bp were ineffective at inducing NOE, and why the magnitude of NOE depended on the dsNHO concentration used. Higher concentrations of dsNHOs could interact with—and eventually saturate—most available KU heterodimers, titrating them away from CRISPR–Cas‐induced DSBs. These freed DSBs would then be repaired primarily by the error‐prone MMEJ pathway. However, the KO efficiency can still increase threefold in the *ku* mutant when CRISPR‐Cas12a RNPs are transfected with dsNHOs (Figure 3A), suggesting that other dsDNA‐binding proteins may also contribute to NOE. Further experiments are required to identify the underlying genes.

The ‘decoy’ model is consistent with exogenous single‐stranded oligonucleotides affecting the localisation of KU proteins in the nucleus of human cells, and the disruption of KU70 foci when nuclei are micro‐irradiated (Yuan et al. 2015). This hypothesis could be investigated further by testing whether the localisation of KU heterodimers to the Cas12a‐mediated DSB is reduced when dsNHOs are co‐delivered (Aparicio et al. 2005).

However, a decoy effect is unlikely to be the only phenomenon contributing to enhancement in KO efficiency because an increase in dsNHO insertions into the targeted DSB was also observed (Figures S1 and S12). These could be explained by the proximity of dsNHOs near the targeted DSB and their subsequent integration by the c‐NHEJ pathway (Figures 1C and 4B), leading to frameshift mutations that boost *FKB12* KO efficiency.

We used non‐phosphorylated oligonucleotides in this study. At the beginning of this work, we omitted 5′ phosphorylation from our oligo design because, in our previous paper, we demonstrated that 5′ phosphorylation of the oligonucleotide used for SSTR (Ferenczi et al. 2021) had no impact on gene‐editing frequency when compared to non‐phosphorylated counterparts. When we later determined that dsNHOs act primarily as decoys and are only infrequently incorporated into the CRISPR cut site, we decided not to modify the oligo design to maintain a low integration frequency at DSBs and minimise off‐target incorporation. We found that 24‐bp dsNHOs were sufficient to induce NOE, and that the KO efficiency did not increase further with lengths up to 96 nt (Figure 2B). This suggests that longer dsDNA may also be effective at triggering NOE in *Chlamydomonas*, as has been observed with salmon sperm DNA in human cell lines (Richardson et al. 2016).

We observed a significant increase in gene knockout efficiency—ranging from fivefold to two orders of magnitude—when dsNHOs were co‐transfected with CRISPR–Cas RNPs, compared to RNPs alone, across all experiments (Figures 1, 4 and Figure S14). These data clearly demonstrate that the addition of dsNHOs can enhance—and often rescue—inefficient CRISPR RNPs, regardless of the nuclease or target locus used. Locus‐specific factors such as chromatin accessibility, gRNA efficacy, local DNA repair dynamics, and the cell cycle (Jain et al. 2024) could each contribute to the observed variability in KO‐boosting efficiency; however, further studies are required to dissect the underlying molecular mechanisms.

Every gene editing technology using exogenous DNA can lead to off‐target integration and consequently unwanted mutations elsewhere in the genome. To circumvent this, we recommend generating three or more independent gene KO lines to verify consequent phenotypes as done previously in published literature (Ferenczi et al. 2021; Sizova et al. 2021). As we have shown significant reduction of dsNHO insertion into the *FKB12* target site in *ku* mutants (Figure 3D,E) and the c‐NHEJ pathway being a major player in gene knockout (Figure 3A), in the future it would be crucial to find an efficient inhibitor of the *C. reinhardtii* KU heterodimer. Such an inhibitor could transiently suppress c‐NHEJ and consequently boost gene editing efficacy in this organism.

## 4 Experimental Procedures

### 4.1 *C. reinhardtii* Strain and Growth Conditions

*C. reinhardtii* were grown on Tris‐acetate‐phosphate (TAP) media (Gorman and Levine 1965) supplemented with 1.5% agar. Asynchronous cells were grown under constant cool fluorescent white light (100 μmol photons·m^−1^·s^−1^) at 27°C, and liquid TAP cultures were shaken at 110 rpm (Stuart SSL1 Orbital Shaker). Experiments using synchronous *C. reinhardtii* cultures were grown in TAP medium in either 14:10 or 12:12 light: dark cycle at 27°C. Cell wall‐less strains containing *cw15* mutation used in this thesis include cell cc‐1883, cc‐3491, cc‐4533, and UVM4 (UV mutant of cc‐4350). The algal growth and physiological experiments involving the *pht4‐7* mutants were performed as described (Tóth et al. 2024).

### 4.2 Purification of Cas12a

We purified LbCas12a from *E. coli* as previously described with minor modifications (Ferenczi et al. 2021). In short, Rosetta (DE3) pLysS cells containing a plasmid with codon‐optimised LbCas12a bearing an N‐terminal MBP‐TEV‐HIS‐NLS tag (plasmid 79 008; Addgene) were induced with 0.5 mM isopropyl‐β‐D‐thiogalactoside (IPTG). Cells were harvested and frozen at −80°C until purification. Thawed cells were resuspended in extraction buffer (Ferenczi et al. 2021) and disrupted using a high‐pressure homogeniser (HPH) Cell Disruptor (Constant Systems) at 25 kPsi or sonication using Soniprep 150 plus disintegrator using 5 cycles of: 10 on, 30 s off, amplitude 3.0. The lysed cells were centrifuged (25 000 × g, 4°C) and the supernatant was transferred to a 50 mL Falcon tube. For affinity purification using beads, 5 mL HisPur cobalt resin (Thermo Scientific) was used according to manufacturer's batch protocol (Ferenczi et al. 2021). Elutions were pooled and concentrated to 200 μL using Vivaspin 50 kDa MWCO (GE Healthcare) or Amicon Ultra 50 kDa MWCO (Millipore), and buffer exchanged into storage buffer. Protein concentration was measured by using Bradford reagent (Sigma) and we aim to achieve final Cas12a protein concentrations between 10 and 20 μg/μL. Single‐use aliquots were snap‐frozen in liquid nitrogen and stored at −80°C.

### 4.3 Guide RNAs (gRNAs) and dsNHOs

gRNAs were purchased from Sigma and Synthego and were resuspended to 200*–*700 μM (Table S1). Non‐5′ phosphorylated single‐stranded oligodeoxynucleotides were purchased from Sigma, IDT, and QIAGEN as dried oligonucleotides and were resuspended to 1 mM (Table S2). dsNHOs were annealed by heating single‐stranded oligodeoxynucleotides to 95°C for 5 min and cooling to 20°C at a rate of −0.3°C/s.

### 4.4 *C. reinhardtii* Transformation for Gene Knockout

*C. reinhardtii* cells were transfected as previously described (Ferenczi et al. 2017). Cultures were grown to approximately 1*–*3 × 10^6^ cells/mL. To form RNPs, Cas12a (0.263 nmol, 50 μg) was incubated at a 1:3 M ratio with gRNA (0.789 nmol) at 37°C for 10*–*15 min. Cells (125 μL, 2.5 × 10^5^ cells) were then mixed with RNPs, oligonucleotides (2.63 nmol) and supplemented with sucrose (40 mM). Electroporation was performed in 4 mm cuvettes (600 V, 50 μF, 200 Ω, 4 mm; Gene Pulser Xcell, Bio‐Rad). Next, the cells were transferred to 5 mL TAP supplemented with sucrose (40 mM) and recovered for 24 h on a shaker. Cells were plated using freshly prepared starch solution (30%, 18). Washes in absolute ethanol twice, thrice in sterile, distilled water, once in TAP, involved vortexing to resuspend the starch, centrifugation (3000 × *g*, 10 s) and decanting. To plate cells, 30*–*250 μL recovered cells was added up to 1 mL with starch solution, mixed well by pipetting and spread equally as 2 × 450 μL onto one TAP plate and one TAP plate supplemented with rapamycin (10 μM). Plated cells were grown under low light (5*–*10 μmol m^−2^ s^−1^) to limit rapamycin photodegradation. After 7 days, plates were imaged and quantified using OpenCFU (v 3.9.0, Geissmann 2013).

### 4.5 *C. reinhardtii* Transformation for Antibiotic Resistance Cassette Knock‐In

Cell cultures were synchronised to a 14:10 h light: dark cycle at 27°C on TAP. The *APHVIII* (Sizova et al. 2002) or *APHVII* (Berthold et al. 2002) DNA cassette (~1.8 and ~1.7 kb respectively) coding for paromomycin and hygromycin resistance respectively was amplified by PCR from pSI103‐1 and pHyg3 plasmid (Table S3, Berthold et al. 2002) using GoTaq Long PCR master mix (Promega) and primers. The PCR products were gel purified using MinElute Gel Extraction kit (Qiagen). Cas12a and Cas9 (25 μg, 0.131 nmol) were assembled as above. For delivery, 62.5 μL synchronised cell culture (1.25 × 10^5^ cells), 40 mM sucrose, and 1 μg *APHVIII* or *APHVII* DNA was mixed with preincubated RNPs in 4‐mm cuvettes and electroporated at 7 h post‐dawn and recovered as above, with subsequent plating on TAP media supplemented with 30 μg/mL paromomycin or 15 μg/mL hygromycin (600 V, 50 μF, 200 Ω) using Gene Pulser Xcell (Bio‐Rad). Colony PCR to confirm antibiotic knock‐in sequence was performed by using the GoTaq Long PCR (Promega, Table S3). Positive knock‐in bands were gel‐purified using MinElute Gel Extraction kit (Qiagen) as per manufacturer's protocol and sent for Sanger sequencing.

### 4.6 *C. reinhardtii* Colony PCR

Colony PCR of *C. reinhardtii* cells was performed one of two ways. Cells were either picked into the Phire Plant Direct PCR kit (Thermo Scientific) dilution buffer and used as per manufacturer's instructions, which 0.5 μL was added into 10 μL GoTaq Long PCR Master Mix (Promega) reactions, respectively. Primers and cycling conditions are provided in Table S3. For PCR clean‐up, PCR reactions were two‐fold diluted, supplemented with exonuclease I (0.18 U/μL; New England Biolabs) and shrimp alkaline phosphatase (0.066 U/μL; New England Biolabs), and incubated at 37°C for 30 min and 80°C for 10 min subsequently for enzyme denaturation. Reactions were sequenced using Sanger sequencing (Azenta Life Sciences). Sequence chromatograms were aligned using Snapgene 5.0.8. Low‐quality sequences (typically Q40/length < 0.3) were excluded together with mixed‐read sequences.

### 4.7 DNA Damaging Agent Assays

OD750 of exponentially growing *C. reinhardtii* cells was measured and adjusted to OD750 of 1 with 30% sterile starch resuspended in TAP. Three 10‐fold serial dilutions were carried out with TAP‐starch, and 10 μL of cell‐starch mixture was spotted onto TAP plates containing relevant DNA‐damaging agents and concentration. Plates were incubated for 7 days under low light (5–10 μmol m^−2^ s^−1^) and photographed.

### 4.8 Amplicon Sequencing Paired‐End Sequencing

Single indexing for each pooled DNA sample was added using Phire Plant Direct PCR Master Mix (Thermo Scientific) with Illumina Nextera forward primer with a unique barcode and adapter and reverse primer with adapters (Table S4). The amplicon PCR was purified using Ampure XP beads (Beckman Coulter) according to the manufacturer's protocol and sent for Azenta Amplicon‐EZ service (2 × 250 bp paired‐end sequencing). Trimmed raw reads from Azenta were separated by barcode using Geneious Prime software and uploaded to CRISPResso2 (Clement et al. 2019).

## Author Contributions

A.M. and Y.P.C. designed the research. Y.P.C., A.F., M.D., C.P.L., A.K., D.T., and A.M. performed the research. A.M. and Y.P.C. analysed the data. Y.P.C. and A.M. wrote the paper with input from all authors.

## Conflicts of Interest

The authors declare no conflicts of interest.

## Supporting information


**Figure S1:** Phenotypic and genotypic analysis of *C. reinhardtii* cells transformed with Cas12a RNP and dsNHOs.
**Figure S2:** Investigating the impact of dsNHO sequence on NOE.
**Figure S3:** *FKB12* KO efficiency for different concentrations of 24‐bp dsNHO with NNNN overhangs co‐delivered with *FKB12* Cas12a RNPs.
**Figure S4:** Analysis of editing events at the *FKB12* cut site of colonies treated with Cas12a RNP alone, or with ssNHOs (A) and dsNHOs (B) with various length.
**Figure S5:** Examining the effect of exogenous RNA on Cas12a‐mediated knockout efficiency at the *FKB12* locus.
**Figure S6:** Analysis of the impact of dsNHO chemical modifications on NOE.
**Figure S7:** Schematic diagram of the decoy hypothesis explaining NOE mechanism in *C. reinhardtii* .
**Figure S8:** Generation of DNA repair mutants in *C. reinhardtii* .
**Figure S9:** Analysis of *kupolq* and *mre11* mutants.
**Figure S10:** Analysing the impact of DNA‐damaging agents on DNA repair mutants.
**Figure S11:** Investigating the effect of dsNHO sequence on NOE in *ku* mutants.
**Figure S12:** Analysis of *C. reinhardtii* cells transformed with Cas9 RNP and dsNHOs.
**Figure S13:** Analysis of gene editing frequency in a population of cells transfected with *PHT4‐7*‐specific Cas12a RNP only.
**Figure S14:** Analysis of the genotype and phenotype of *pht4‐7* mutants.
**Figure S15:** Predicted secondary structure of the 127‐nt long ssNHO (25).

**Table S1:** List of SpCas9 and LbCas12a gRNAs used to target endogenous reporter genes.
**Table S2:** List of non‐homologous oligonucleotides (NHOs) used, including the descriptions of their use and related figures.
**Table S3:** List of primers used to amplify antibiotic selection markers and endogenous CRISPR target locus.
**Table S4:** List of amplicon primers used for deep amplicon sequencing.
**Table S5:** List of templates and primer used for in vitro transcription of #2 RNA and #7–3 RNA.
**Table S6:** Tukey's post hoc test statistical summary of one‐way ANOVA results when comparing more than two groups. ANOVA results are reported for figures comparing between two groups.
**Table S7:** Raw knockout editing values and mutation type frequency.

## Data Availability

The data that support the findings of this study are available on request from the corresponding author. The data are not publicly available due to privacy or ethical restrictions.

## References

[pbi70548-bib-0001] Akella, S. , X. Ma , R. Bacova , et al. 2021. “Co‐Targeting Strategy for Precise, Scarless Gene Editing With CRISPR/Cas9 and Donor ssODNs in *Chlamydomonas* .” Plant Physiology 187, no. 4: 2637–2655. 34618092 10.1093/plphys/kiab418 PMC8644747

[pbi70548-bib-0002] Angstenberger, M. , F. de Signori , V. Vecchi , L. Dall'Osto , and R. Bassi . 2020. “Cell Synchronization Enhances Nuclear Transformation and Genome Editing via Cas9 Enabling Homologous Recombination in *Chlamydomonas reinhardtii* .” ACS Synthetic Biology 9, no. 10: 2840–2850. 32916053 10.1021/acssynbio.0c00390 PMC8011982

[pbi70548-bib-0003] Aparicio, O. , J. V. Geisberg , E. Sekinger , A. Yang , Z. Moqtaderi , and K. Struhl . 2005. “Chromatin Immunoprecipitation for Determining the Association of Proteins With Specific Genomic Sequences In Vivo.” Current Protocols in Molecular Biology 69, no. 1: 21–23. 10.1002/0471142727.mb2103s69 18265358

[pbi70548-bib-0004] Baek, K. , D. H. Kim , J. Jeong , et al. 2016. “DNA‐Free Two‐Gene Knockout in *Chlamydomonas reinhardtii* via CRISPR‐Cas9 Ribonucleoproteins.” Scientific Reports 6, no. 1: 30620. 27466170 10.1038/srep30620 PMC4964356

[pbi70548-bib-0005] Berthold, P. , R. Schmitt , and W. Mages . 2002. “An Engineered *Streptomyces hygroscopicus* Aph 7′ Gene Mediates Dominant Resistance Against Hygromycin B in *Chlamydomonas reinhardtii* .” Protist 153, no. 4: 401–412. 12627869 10.1078/14344610260450136

[pbi70548-bib-0006] Black, S. J. , E. Kashkina , T. Kent , and R. T. Pomerantz . 2016. “DNA Polymerase θ: A Unique Multifunctional End‐Joining Machine.” Genes 7, no. 9: 67. 27657134 10.3390/genes7090067 PMC5042397

[pbi70548-bib-0007] Camperi, J. , M. Moshref , L. Dai , and H. Y. Lee . 2021. “Physicochemical and Functional Characterization of Differential CRISPR‐Cas9 Ribonucleoprotein Complexes.” Analytical Chemistry 94, no. 2: 1432–1440. 34958212 10.1021/acs.analchem.1c04795

[pbi70548-bib-0008] Chang, H. H. , N. R. Pannunzio , N. Adachi , and M. R. Lieber . 2017. “Non‐Homologous DNA End Joining and Alternative Pathways to Double‐Strand Break Repair.” Nature Reviews Molecular Cell Biology 18, no. 8: 495–506. 28512351 10.1038/nrm.2017.48 PMC7062608

[pbi70548-bib-0009] Clement, K. , H. Rees , M. C. Canver , et al. 2019. “CRISPResso2 Provides Accurate and Rapid Genome Editing Sequence Analysis.” Nature Biotechnology 37, no. 3: 224–226. 10.1038/s41587-019-0032-3 PMC6533916 30809026

[pbi70548-bib-0010] Clerici, M. , D. Mantiero , I. Guerini , G. Lucchini , and M. P. Longhese . 2008. “The Yku70–Yku80 Complex Contributes to Regulate Double‐Strand Break Processing and Checkpoint Activation During the Cell Cycle.” EMBO Reports 9, no. 8: 810–818. 18600234 10.1038/embor.2008.121 PMC2515202

[pbi70548-bib-0011] Conant, D. , T. Hsiau , N. Rossi , et al. 2022. “Inference of CRISPR Edits From Sanger Trace Data.” CRISPR Journal 5, no. 1: 123–130. 35119294 10.1089/crispr.2021.0113

[pbi70548-bib-0012] Crinelli, R. , M. Bianchi , L. Gentilini , and M. Magnani . 2002. “Design and Characterization of Decoy Oligonucleotides Containing Locked Nucleic Acids.” Nucleic Acids Research 30, no. 11: 2435–2443. 12034831 10.1093/nar/30.11.2435 PMC117200

[pbi70548-bib-0013] Dhokane, D. , B. Bhadra , and S. Dasgupta . 2020. “CRISPR Based Targeted Genome Editing of *Chlamydomonas reinhardtii* Using Programmed Cas9‐gRNA Ribonucleoprotein.” Molecular Biology Reports 47: 8747–8755. 33074412 10.1007/s11033-020-05922-5

[pbi70548-bib-0015] Falzon, M. , J. W. Fewell , and E. L. Kuff . 1993. “EBP‐80, a Transcription Factor Closely Resembling the Human Autoantigen Ku, Recognizes Single‐ to Double‐Strand Transitions in DNA.” Journal of Biological Chemistry 268, no. 14: 10546–10552. 8486707

[pbi70548-bib-0016] Ferenczi, A. , Y. P. Chew , E. Kroll , C. von Koppenfels , A. Hudson , and A. Molnar . 2021. “Mechanistic and Genetic Basis of Single‐Strand Templated Repair at Cas12a‐Induced DNA Breaks in *Chlamydomonas reinhardtii* .” Nature Communications 12, no. 1: 6751. 10.1038/s41467-021-27004-1 PMC8604939 34799578

[pbi70548-bib-0017] Ferenczi, A. , M. Fellbaum , Y. P. Chew , C. Kidner , and A. Molnar . 2024. “Comparison of CRISPR/Cas9 and Cas12a for Gene Editing in *Chlamydomonas reinhardtii* .” Algal Research 84: 103796.

[pbi70548-bib-0018] Ferenczi, A. , D. E. Pyott , A. Xipnitou , and A. Molnar . 2017. “Efficient Targeted DNA Editing and Replacement in *Chlamydomonas reinhardtii* Using Cpf1 Ribonucleoproteins and Single‐Stranded DNA.” Proceedings of the National Academy of Sciences 114, no. 51: 13567–13572. 10.1073/pnas.1710597114 PMC5754772 29208717

[pbi70548-bib-0019] Geissmann, Q. 2013. “OpenCFU, a New Free and Open‐Source Software to Count Cell Colonies and Other Circular Objects.” PLoS One 8, no. 2: e54072. 23457446 10.1371/journal.pone.0054072 PMC3574151

[pbi70548-bib-0020] Gorman, D. S. , and R. P. Levine . 1965. “Cytochrome f and Plastocyanin: Their Sequence in the Photosynthetic Electron Transport Chain of *Chlamydomonas reinhardtii* .” Proceedings of the National Academy of Sciences 54, no. 6: 1665–1669. 10.1073/pnas.54.6.1665 PMC300531 4379719

[pbi70548-bib-0021] Greiner, A. , S. Kelterborn , H. Evers , G. Kreimer , I. Sizova , and P. Hegemann . 2017. “Targeting of Photoreceptor Genes in *Chlamydomonas reinhardtii* via Zinc‐Finger Nucleases and CRISPR/Cas9.” Plant Cell 29, no. 10: 2498–2518. 28978758 10.1105/tpc.17.00659 PMC5774583

[pbi70548-bib-0022] Hosoya, T. , H. Takeuchi , Y. Kanesaka , et al. 1999. “Sequence‐Specific Inhibition of a Transcription Factor by Circular Dumbbell DNA Oligonucleotides.” FEBS Letters 461, no. 3: 136–140. 10567684 10.1016/s0014-5793(99)01450-7

[pbi70548-bib-0023] Jain, S. , G. Xun , and H. Zhao . 2024. “Impact of Chromatin Organization and Epigenetics on CRISPR‐Cas and TALEN Genome Editing.” ACS Synthetic Biology 13, no. 10: 3056–3068. 39315937 10.1021/acssynbio.4c00099

[pbi70548-bib-0024] Jiang, W. Z. , and D. P. Weeks . 2017. “A Gene‐Within‐a‐Gene Cas9/sgRNA Hybrid Construct Enables Gene Editing and Gene Replacement Strategies in *Chlamydomonas reinhardtii* .” Algal Research 26: 474–480.

[pbi70548-bib-0025] Kelso, A. A. , F. W. Lopezcolorado , R. Bhargava , and J. M. Stark . 2019. “Distinct Roles of RAD52 and POLQ in Chromosomal Break Repair and Replication Stress Response.” PLoS Genetics 15, no. 8: e1008319. 31381562 10.1371/journal.pgen.1008319 PMC6695211

[pbi70548-bib-0027] Kim, J. , S. Lee , K. Baek , and E. Jin . 2020. “Site‐Specific Gene Knock‐Out and On‐Site Heterologous Gene Overexpression in *Chlamydomonas reinhardtii* via a CRISPR‐Cas9‐Mediated Knock‐In Method.” Frontiers in Plant Science 11: 306. 32265959 10.3389/fpls.2020.00306 PMC7099044

[pbi70548-bib-0028] Kirst, H. , J. G. García‐Cerdán , A. Zurbriggen , and A. Melis . 2012. “Assembly of the Light‐Harvesting Chlorophyll Antenna in the Green Alga *Chlamydomonas reinhardtii* Requires Expression of the TLA2‐CpFTSY Gene.” Plant Physiology 158, no. 2: 930–945. 22114096 10.1104/pp.111.189910 PMC3271779

[pbi70548-bib-0030] Nievergelt, A. P. , D. R. Diener , A. Bogdanova , T. Brown , and G. Pigino . 2023. “Efficient Precision Editing of Endogenous *Chlamydomonas reinhardtii* Genes With CRISPR‐Cas.” Cell Reports Methods 3, no. 8: 100562. 37671018 10.1016/j.crmeth.2023.100562 PMC10475843

[pbi70548-bib-0031] Picariello, T. , Y. Hou , T. Kubo , et al. 2020. “TIM, a Targeted Insertional Mutagenesis Method Utilizing CRISPR/Cas9 in *Chlamydomonas reinhardtii* .” PLoS One 15, no. 5: e0232594. 32401787 10.1371/journal.pone.0232594 PMC7219734

[pbi70548-bib-0033] Richardson, C. D. , G. J. Ray , N. L. Bray , and J. E. Corn . 2016. “Non‐Homologous DNA Increases Gene Disruption Efficiency by Altering DNA Repair Outcomes.” Nature Communications 7, no. 1: 12463. 10.1038/ncomms12463 PMC4992056 27530320

[pbi70548-bib-0034] Rodgers, K. , and M. McVey . 2016. “Error‐Prone Repair of DNA Double‐Strand Breaks.” Journal of Cellular Physiology 231, no. 1: 15–24. 26033759 10.1002/jcp.25053 PMC4586358

[pbi70548-bib-0035] Ross, I. L. , H. P. Le , S. Budiman , et al. 2024. “A Cyclical Marker System Enables Indefinite Series of Oligonucleotide‐Directed Gene Editing in *Chlamydomonas reinhardtii* .” Plant Physiology 196, no. 4: 2330–2345. 39179421 10.1093/plphys/kiae427 PMC11637769

[pbi70548-bib-0036] Scaife, M. A. , G. T. Nguyen , J. Rico , D. Lambert , K. E. Helliwell , and A. G. Smith . 2015. “Establishing *Chlamydomonas reinhardtii* as an Industrial Biotechnology Host.” The Plant Journal 82, no. 3: 532–546. 25641561 10.1111/tpj.12781 PMC4515103

[pbi70548-bib-0037] Shin, S. E. , J. M. Lim , H. G. Koh , et al. 2016. “CRISPR/Cas9‐Induced Knockout and Knock‐In Mutations in *Chlamydomonas reinhardtii* .” Scientific Reports 6, no. 1: 27810. 27291619 10.1038/srep27810 PMC4904240

[pbi70548-bib-0038] Sizova, I. , S. Kelterborn , V. Verbenko , S. Kateriya , and P. Hegemann . 2021. “ *Chlamydomonas* POLQ Is Necessary for CRISPR/Cas9‐Mediated Gene Targeting.” G3: Genes, Genomes, Genetics 11, no. 7: jkab114. 33836052 10.1093/g3journal/jkab114 PMC8495919

[pbi70548-bib-0039] Sizova, I. A. , P. Hegemann , M. Furmann , and V. N. Danilenko . 2002. “ *Streptomyces rimosus* Aminoglycoside 3′‐Phosphotransferase VIII: Comparisons With Aminoglycoside 3′‐Phosphotransferases of Aminoglycoside‐Producing Strains and With Eukaryotic Protein Kinases.” Molecular Biology 36: 18–25. 11862708

[pbi70548-bib-0040] Strohkendl, I. , F. A. Saifuddin , J. R. Rybarski , I. J. Finkelstein , and R. Russell . 2018. “Kinetic Basis for DNA Target Specificity of CRISPR‐Cas12a.” Molecular Cell 71, no. 5: 816–824. 30078724 10.1016/j.molcel.2018.06.043 PMC6679935

[pbi70548-bib-0041] Syed, A. , and J. A. Tainer . 2018. “The MRE11–RAD50–NBS1 Complex Conducts the Orchestration of Damage Signaling and Outcomes to Stress in DNA Replication and Repair.” Annual Review of Biochemistry 87, no. 1: 263–294. 10.1146/annurev-biochem-062917-012415 PMC6076887 29709199

[pbi70548-bib-0042] Thierry, A. R. , and A. Dritschilo . 1992. “Intracellular Availability of Unmodified, Phosphorothioated and Liposomally Encapsulated Oligodeoxynucleotides for Antisense Activity.” Nucleic Acids Research 20, no. 21: 5691–5698. 1454532 10.1093/nar/20.21.5691 PMC334404

[pbi70548-bib-0043] Tóth, D. , S. Kuntam , Á. Ferenczi , et al. 2024. “Chloroplast Phosphate Transporter CrPHT4‐7 Regulates Phosphate Homeostasis and Photosynthesis in Chlamydomonas.” Plant Physiology 194, no. 3: 1646–1661. 37962583 10.1093/plphys/kiad607 PMC10904345

[pbi70548-bib-0044] Walker, J. R. , R. A. Corpina , and J. Goldberg . 2001. “Structure of the Ku Heterodimer Bound to DNA and Its Implications for Double‐Strand Break Repair.” Nature 412, no. 6847: 607–614. 11493912 10.1038/35088000

[pbi70548-bib-0045] Xue, J. H. , G. D. Chen , F. Hao , et al. 2019. “A Vitamin‐C‐Derived DNA Modification Catalysed by an Algal TET Homologue.” Nature 569, no. 7757: 581–585. 31043749 10.1038/s41586-019-1160-0 PMC6628258

[pbi70548-bib-0046] Yaneva, M. , T. Kowalewski , and M. R. Lieber . 1997. “Interaction of DNA‐Dependent Protein Kinase With DNA and With Ku: Biochemical and Atomic‐Force Microscopy Studies.” EMBO Journal 16, no. 16: 5098–5112. 9305651 10.1093/emboj/16.16.5098 PMC1170144

[pbi70548-bib-0047] Yuan, Y. , S. Britton , C. Delteil , et al. 2015. “Single‐Stranded DNA Oligomers Stimulate Error‐Prone Alternative Repair of DNA Double‐Strand Breaks Through Hijacking Ku Protein.” Nucleic Acids Research 43, no. 21: 10264–10276. 26350212 10.1093/nar/gkv894 PMC4666393

[pbi70548-bib-0048] Zelensky, A. N. , J. Schimmel , H. Kool , R. Kanaar , and M. Tijsterman . 2017. “Inactivation of Pol θ and C‐NHEJ Eliminates Off‐Target Integration of Exogenous DNA.” Nature Communications 8, no. 1: 66. 10.1038/s41467-017-00124-3 PMC5501794 28687761

[pbi70548-bib-0049] Zetsche, B. , J. S. Gootenberg , O. O. Abudayyeh , et al. 2015. “Cpf1 Is a Single RNA‐Guided Endonuclease of a Class 2 CRISPR‐Cas System.” Cell 163, no. 3: 759–771. 26422227 10.1016/j.cell.2015.09.038 PMC4638220

