# Supplementary material for: Enhancing CRISPR/Cas‐Mediated Gene Knockout With Short Non‐Homologous Oligonucleotides
Source: Plant Biotechnol J. 2026 Feb 22;24(6):3742–53. doi: 10.1111/pbi.70548 (PMC13205664; doi:10.1111/pbi.70548)
Supplement: Supplementary file 1 — Figure S1: Phenotypic and genotypic analysis of C. reinhardtii cells transformed with Cas12a RNP and dsNHOs. Figure S2: Investigating the impact of dsNHO sequence on NOE. Figure S3: FKB12 KO efficiency for different concentrations of 24‐bp dsNHO with NNNN overhangs co‐delivered with FKB12 Cas12a RNPs. Figure S4: Analysis of editing events at the FKB12 cut site of colonies treated with Cas12a RNP alone, or with ssNHOs (A) and dsNHOs (B) with various length. Figure S5: Examining the effect of exogenous RNA on Cas12a‐mediated knockout efficiency at the FKB12 locus. Figure S6: Analysis of the impact of dsNHO chemical modifications on NOE. Figure S7: Schematic diagram of the decoy hypothesis explaining NOE mechanism in C. reinhardtii . Figure S8: Generation of DNA repair mutants in C. reinhardtii . Figure S9: Analysis of kupolq and mre11 mutants. Figure S10: Analysing the impact of DNA‐damaging agents on DNA repair mutants. Figure S11: Investigating the effect of dsNHO sequence on NOE in ku mutants. Figure S12: Analysis of C. reinhardtii cells transformed with Cas9 RNP and dsNHOs. Figure S13: Analysis of gene editing frequency in a population of cells transfected with PHT4‐7‐specific Cas12a RNP only. Figure S14: Analysis of the genotype and phenotype of pht4‐7 mutants. Figure S15: Predicted secondary structure of the 127‐nt long ssNHO (25). [file PBI-24-3742-s001.docx]

**Supplementary figures
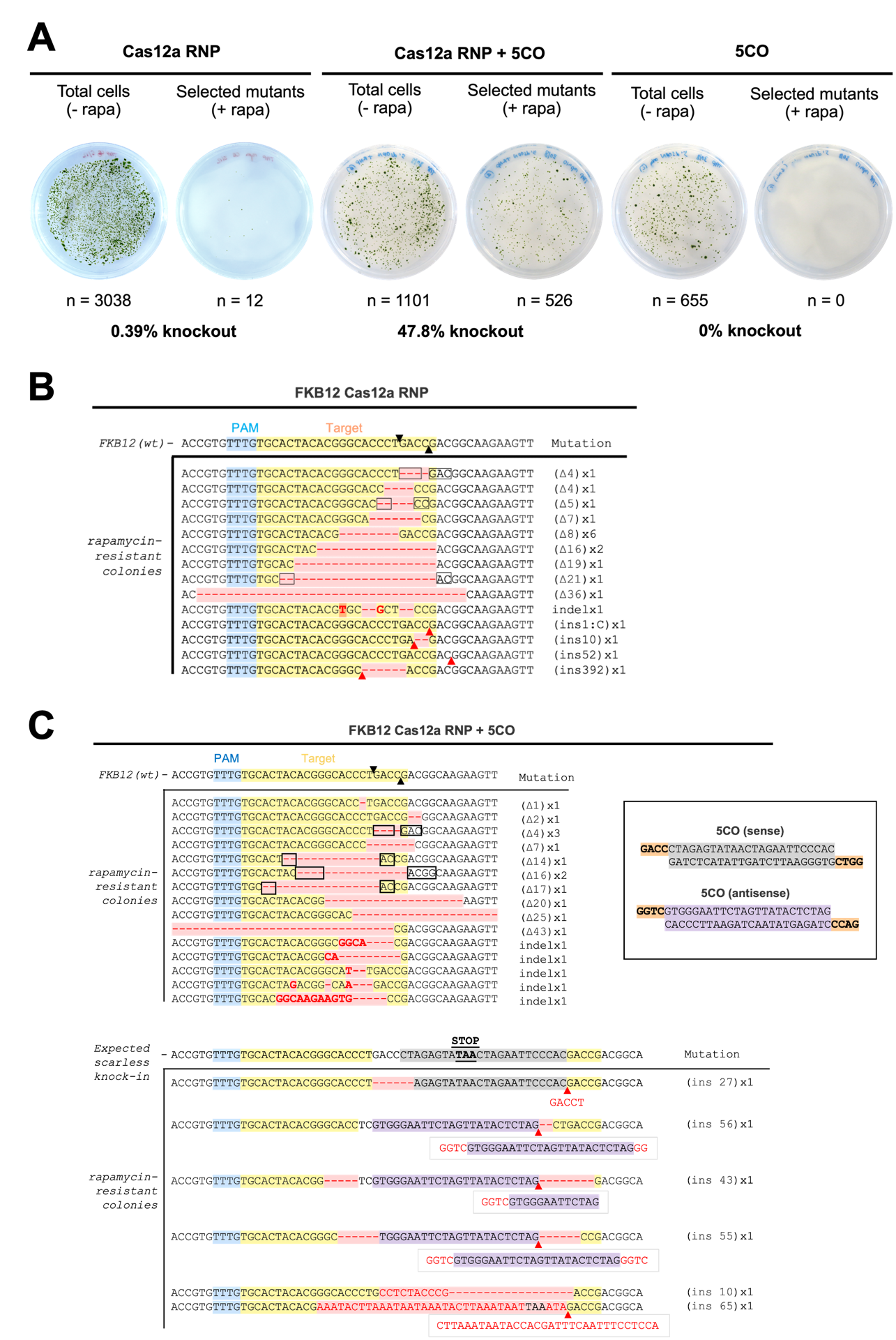

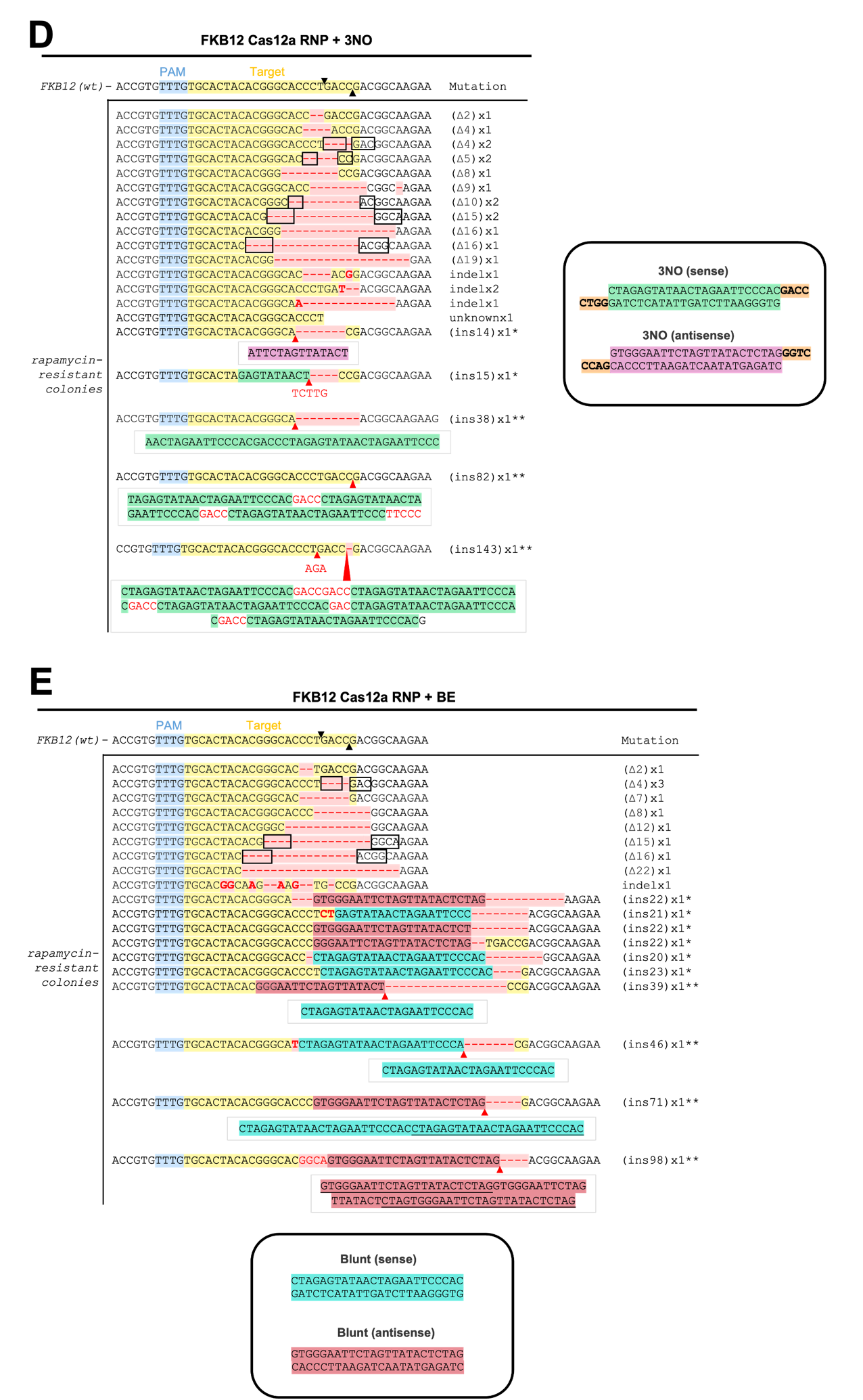
**

# Figure S1. (A) Cells transformed with Cas12a RNP and/or 5CO were diluted with 30% starch and spread 50:50 onto solid growth media with and without 10 μM rapamycin (rapa). *FKB12* KO efficiency (%) is calculated as number of cells growing on TAP with rapa per total number of cells growing on TAP without rapa. (B) *FKB12* locus from randomly selected rapamycin-resistant colonies treated with Cas12a RNP alone (B) or alongside (C) 5CO, (D) 3NO or (E) BE, were sequenced. Black triangles indicate the expected Cas12a-mediated cleavage site, red triangles indicate insertion sites, and red highlights indicates sequence deviation from the WT sequence (top). Black boxes indicate potential microhomology-mediated deletions. Deletion (Δ) and insertion size (base pairs) are shown to the right of the sequence alongside the count of sequence observations. (B) The expected scarless 5CO knock-in sequence is indicated and the expected premature stop codon is highlighted (black, bold and underlined text). 5CO was seen to be inserted in two different orientations indicated in the black box. The identity of insertion sequences is shown in light grey boxes. Single and concatenated dsODN insertions are indicated by asterisks (* and ** respectively).

# **
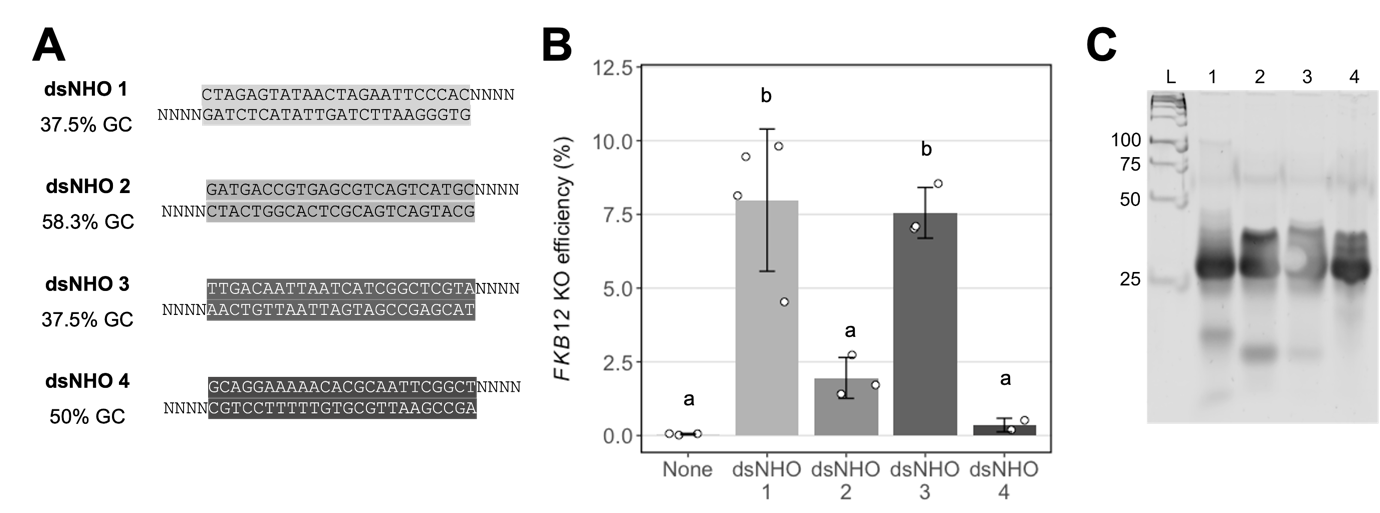
**

**Figure S2**. (A) Schematic diagram of 24-bp dsNHO with different sequences and GC content. Complementary oligonucleotides were annealed by heating to 95°C for 5 minutes and slowly cooled to room temperature. (B) *FKB12* KO efficiency between cells treated with Cas12a RNP without or with four different sequences of dsNHO (2≤n≤4). Bars labelled with different letters were significantly different (p < 0.01) according to one-way ANOVA test. The error bars represent the standard deviation. (C) 15% TBE PAGE GEL showing 30 µM dsNHO 1 – 4 (#1 - #4) after annealing forward and reverse oligos at same molarity for 95°C for 5 minutes. L: GeneRuler Low Range DNA Ladder (Thermo Scientific) is shown in base pairs.

# **
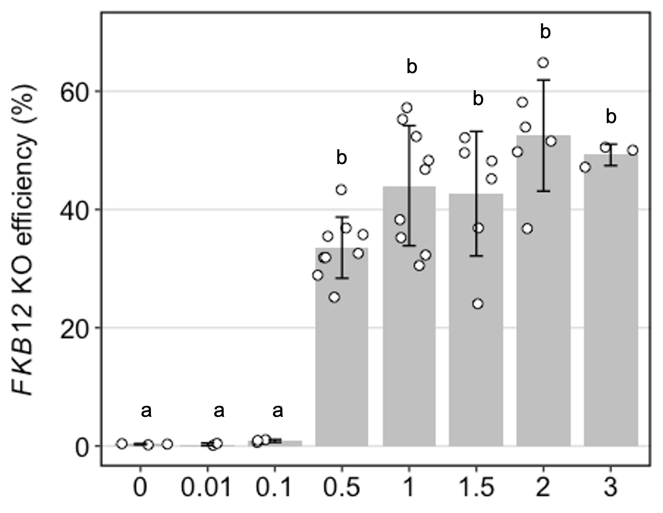
**

**Figure S3**. *FKB12* KO efficiency for different concentrations of 24-bp dsNHO with NNNN overhangs co-delivered with *FKB12* Cas12a RNPs. Bars labelled with different letters were significantly different (p < 0.001) according to one-way ANOVA test. The error bars represent the standard deviation.

# **
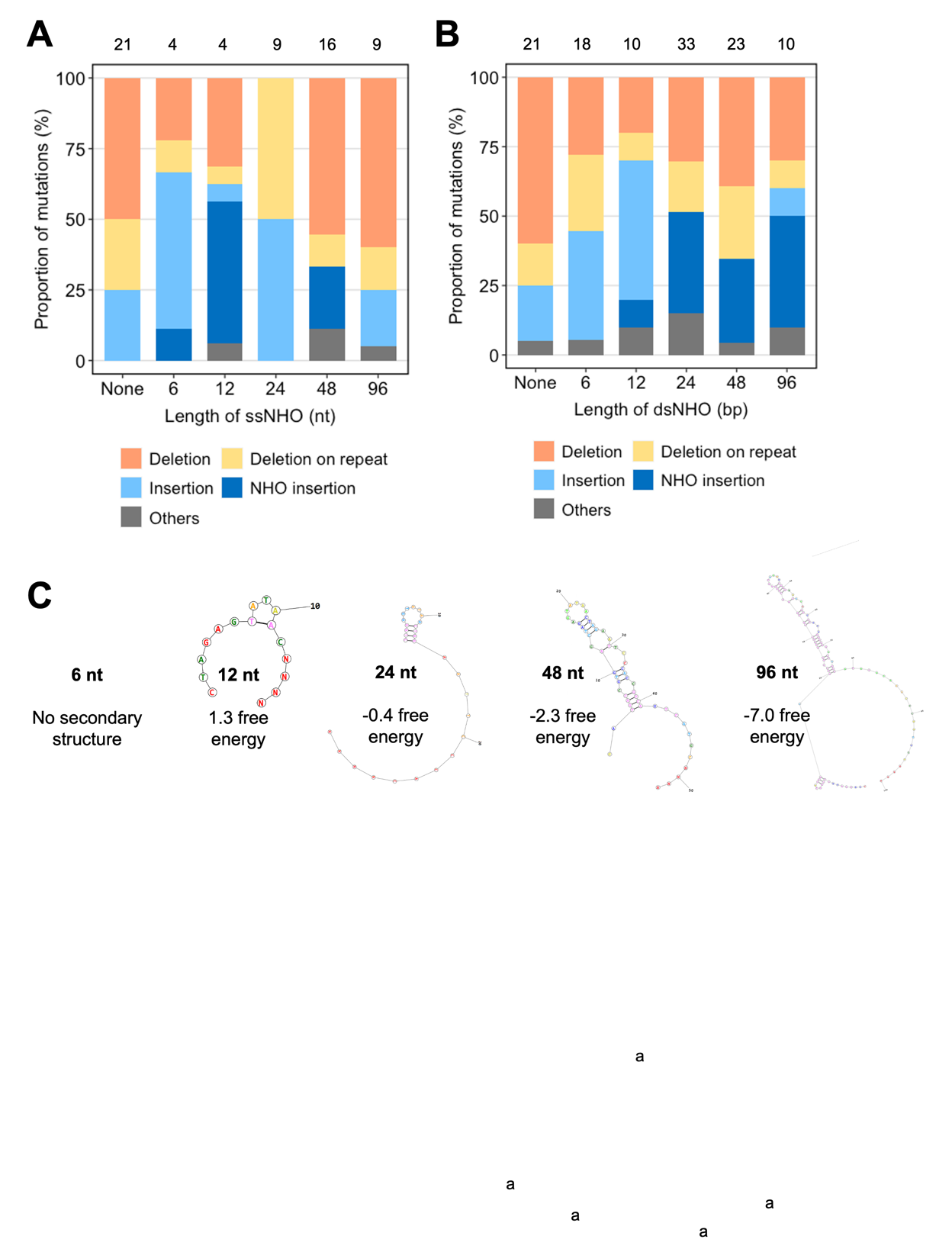
**

**Figure S4**. Analysis of editing events at the *FKB12* cut site of colonies treated with Cas12a RNP alone, or with ssNHOs (A) and dsNHOs (B) with various length. The number of successful Sanger sequences analysed are indicated at the top of the bars. The ‘Deletion on repeat’ category represents sequences that potentially have microhomology-mediated deletions whereas ‘Deletion’ indicates deletions that do not. ‘Oligo insertion’ represents any sequences that have homology to dsNHOs and ‘Insertion’ represents sequences that do not have homology. The ‘Others’ category is a mixture of mismatches and unknown sequences that end abruptly in the Sanger chromatogram. (C) Predicted secondary structure and free energy of ssNHOs using RNAstructure software (Reuter and Mathews, 2010). The total length of single-stranded oligodeoxynucleotide is indicated.

# **
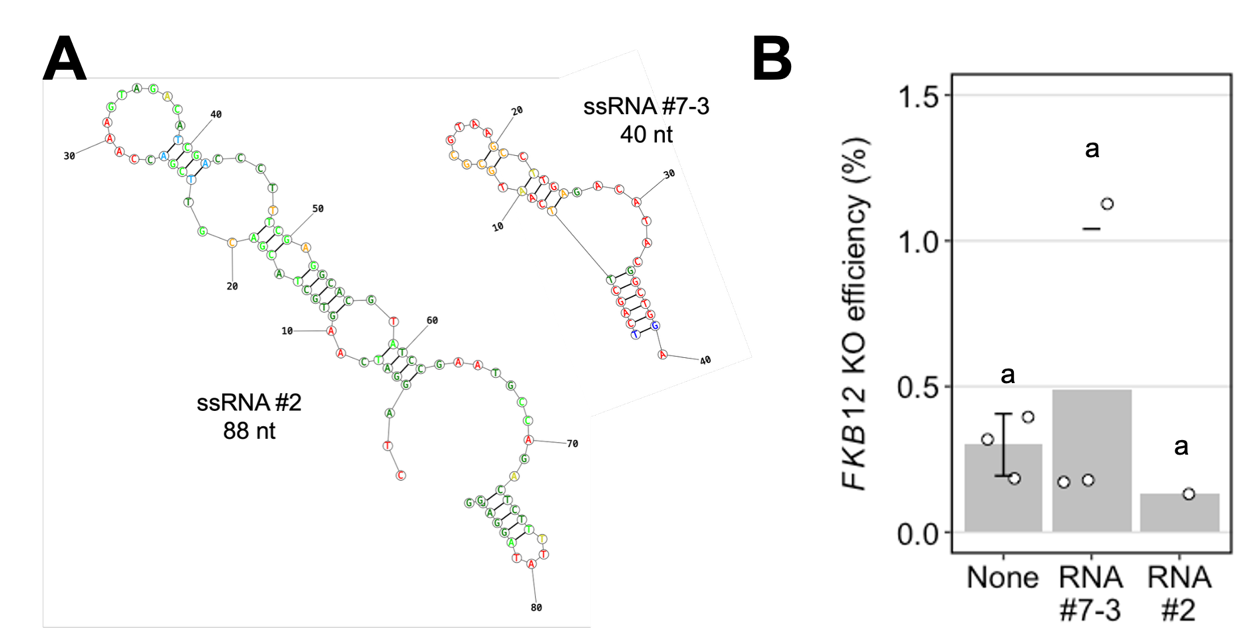
**

**Figure S5**. (A) Predicted secondary structure of single-stranded RNAs (ssRNA), RNA #7-3 and RNA #2, using RNAstructure software. The total length of ssRNA is indicated. These ssRNAs are derived from an expired patent (Dynan and Yoo, 2024) with its indicated length, and *in vitro* transcribed using primers in Supplementary Table 5. (B) *FKB12* KO efficiency of cells transformed with *FKB12* Cas12a RNPs and/or RNA #7-3 or RNA #2 (1≤n≤3). Bars labelled with different letters were significantly different (p < 0.05) according to one-way ANOVA test. The error bars represent the standard deviation.

# **
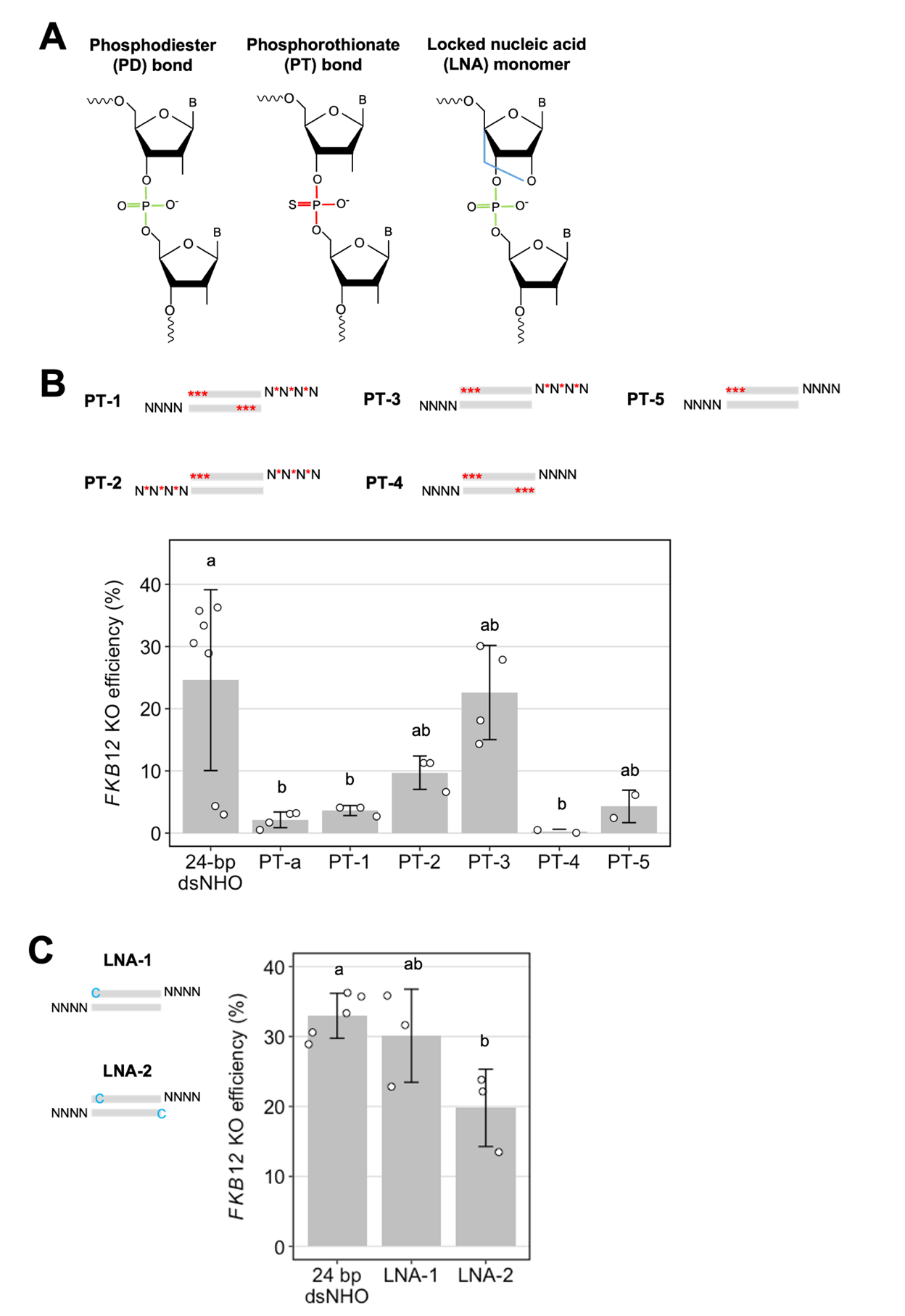
**

**Figure S6**. (A) Schematic diagram of the phosphodiester (PD) backbone bond, a phosphonothioate (PT) chemical modification and locked nucleic acid (LNA) monomer. ‘B’ indicates a base. (B) The impact of PT modifications at termini of non-homologous oligonucleotides (NHO) on *FKB12* KO efficacy in *C. reinhardtii* cc-1883 (2≤n≤7). (C) *FKB12* KO efficiency (3≤n≤5) between cells co-transfected with Cas12a RNPs and 24-bp dsNHO, and dsNHOs with LNA at one 5’ end of dsNHO (LNA-1) or both (LNA-2). Bars labelled with different letters were significantly different (p < 0.05) according to one-way ANOVA test. The error bars represent the standard deviation.

**
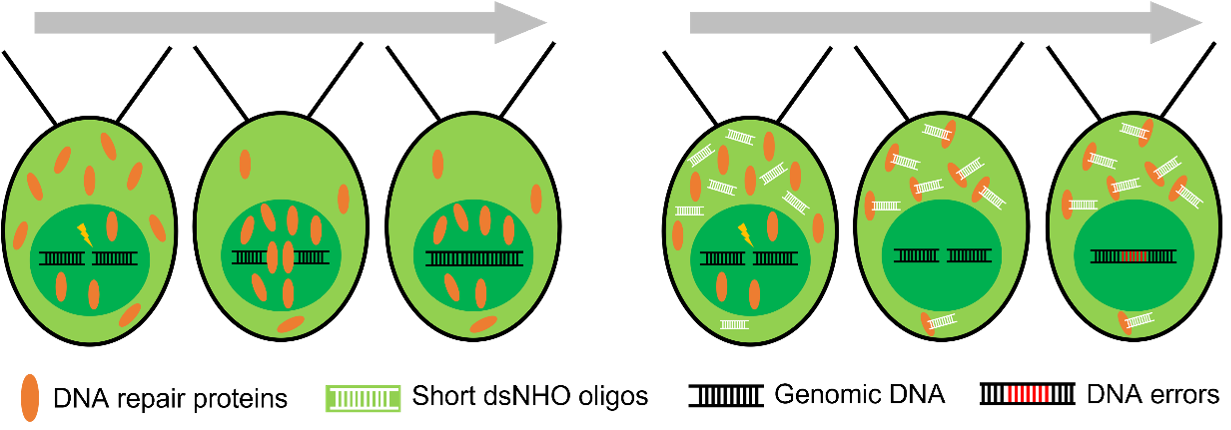
**

**Figure S7**. Schematic diagram of the decoy hypothesis explaining NOE mechanism in *C. reinhardtii*. Left: When a double-stranded break (DSB) occurs in the genome, DNA repair proteins (orange) are localised to the nucleus (dark green) to carry out DNA repair. Right: When exogenous NHO (white) are introduced into the cell, DNA repair proteins (orange) can bind to NHO. NHO act as a decoy, sequestering DNA repair proteins away from the generated DSB, consequently leading to error-prone repair (red) at the break site.

**
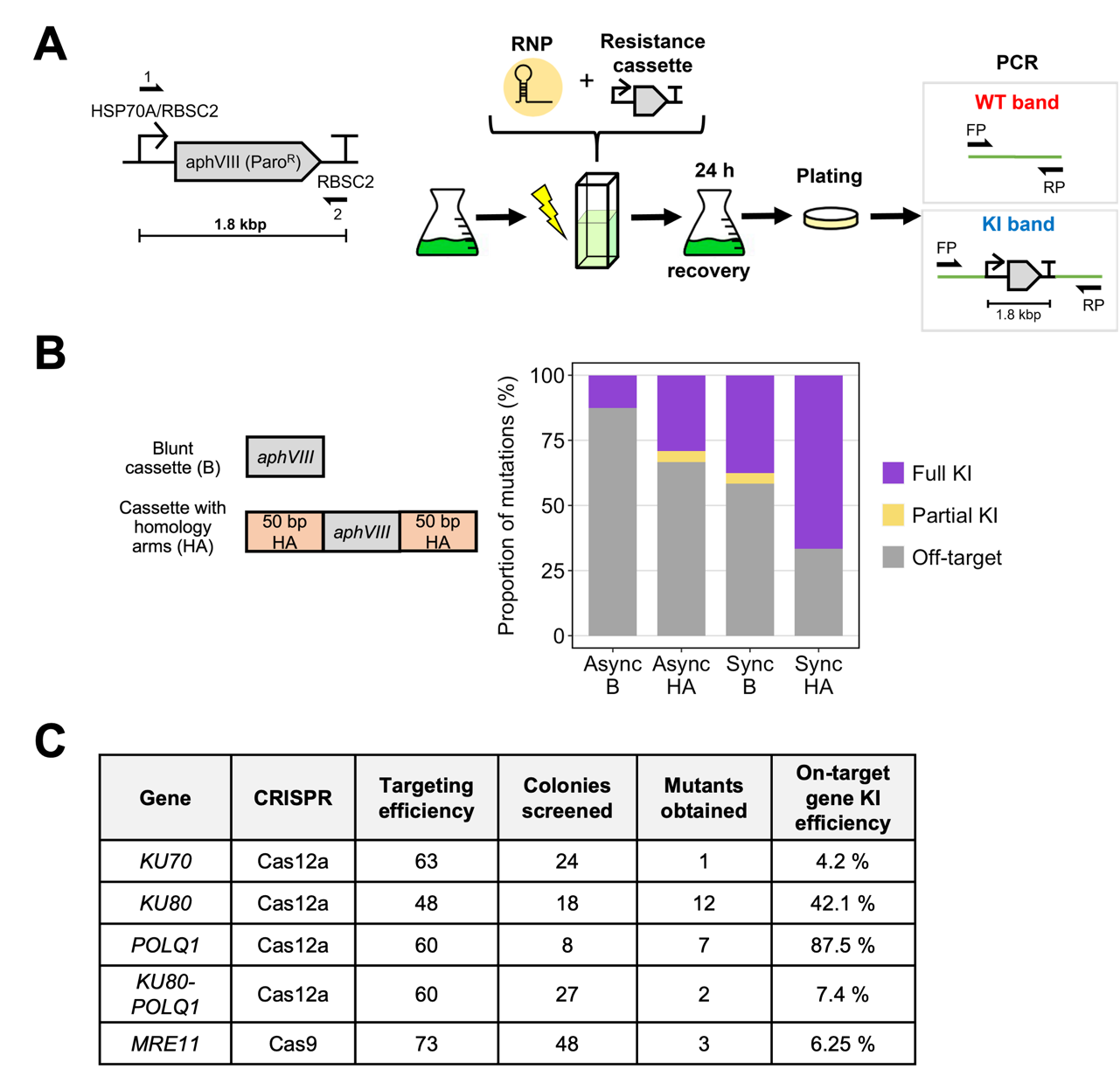
**

**Figure S8**. (A) Schematic of Ab^R^ knock-in experimental set up. Primers were used to amplify the paromomycin resistance (paro^R^) gene *aphVIII* cassette from pSI103 plasmid for transformation. 12:12 synchronised *C. reinhardtii* cc-1883 cells were electroporated with Cas12a or Cas9 RNP targeting the gene-of-interest alongside the paro^R^ cassette, recovered for 24 hours, and plated onto TAP supplemented with paromomycin. Resistant colonies, alongside cc1883 wild-type control, were screened for positive on-target *aphVIII* knock-in by using gene-specific forward (FP) and reverse primers (RP). Any bands that are bigger than 1.8 kb or WT band are considered positive mutants. (B) On-and off-target knock-in was determined for asynchronous (Async) or synchronous (Sync) cc-1883 transformed with Ku80 Cas12a RNPs and 1 µg blunt (B) *aphVIII* cassette or *aphVIII* cassette with 50-bp homology arm (HA) homologous to regions around the Cas12a-mediated DSB at *KU80* locus. On-target gene knock-in efficiency was calculated (n=24) as number of mutants with full or partial *aphVIII* insertion obtained per total number of colonies screened via PCR. (C) On-target gene knock-in efficiency obtained for several target locus using synchronous cc-1883 cultures that were transformed with Cas12a or Cas9 RNP with 1 µg blunt *aphVIII* cassette. Predicted targeting efficiency scores for Cas12a and Cas9 was determined by DeepCpf1 (Kim et al. 2018) and CRISPRscan, respectively (Moreno‐Mateos et al. 2015).

**
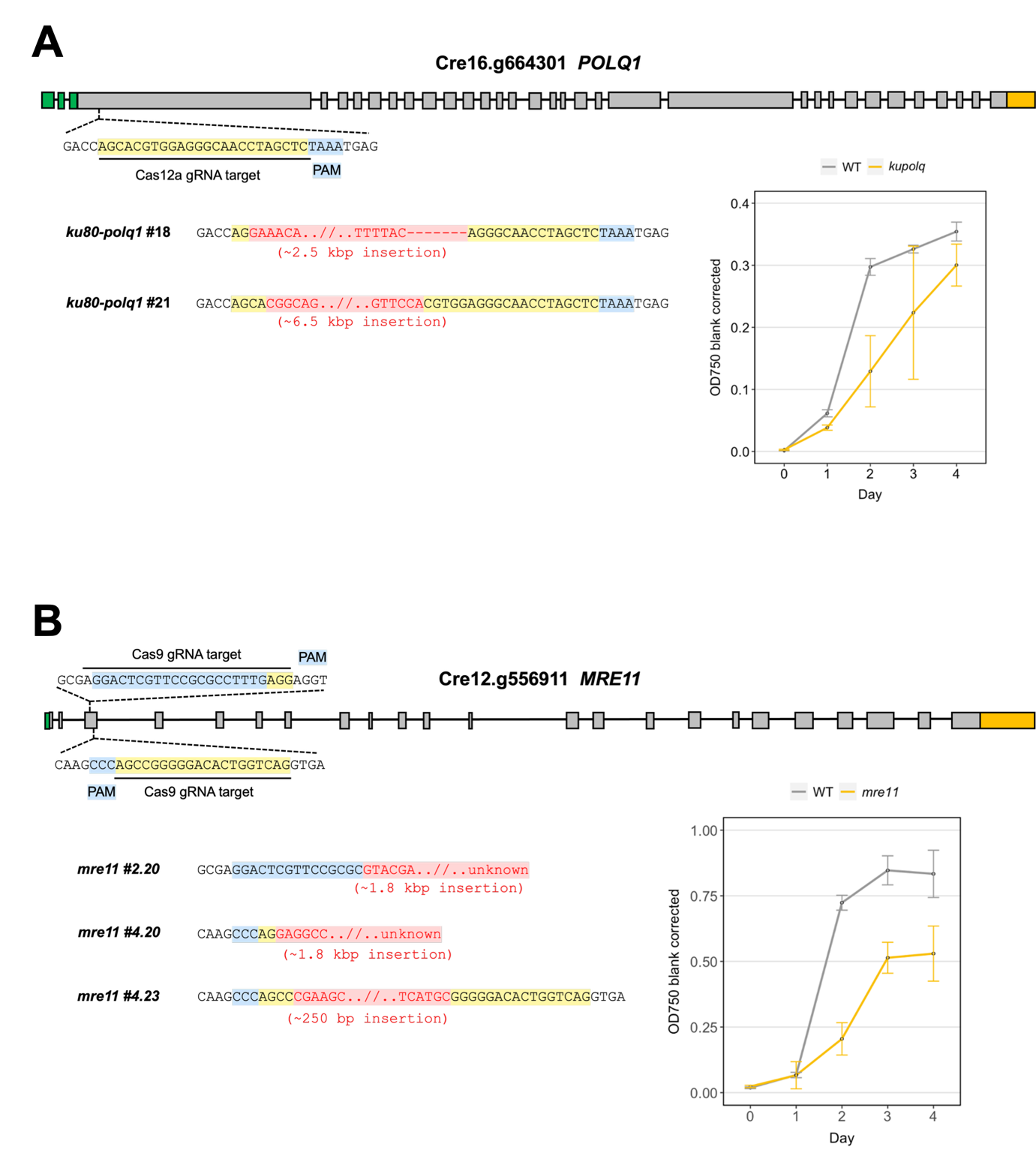
**

**Figure S9**. Two *kupolq* double mutants lines (A) and three *mre11* mutant lines were generated. The independent mutant lines (named after hashtag #) have a different gene-*aphvIII* junction sequences. Growth curve of WT and mutants were obtained using OD750 over four days. The *polq* and *ku* mutant lines used for analysis were previously published (Ferenczi et al. 2021).


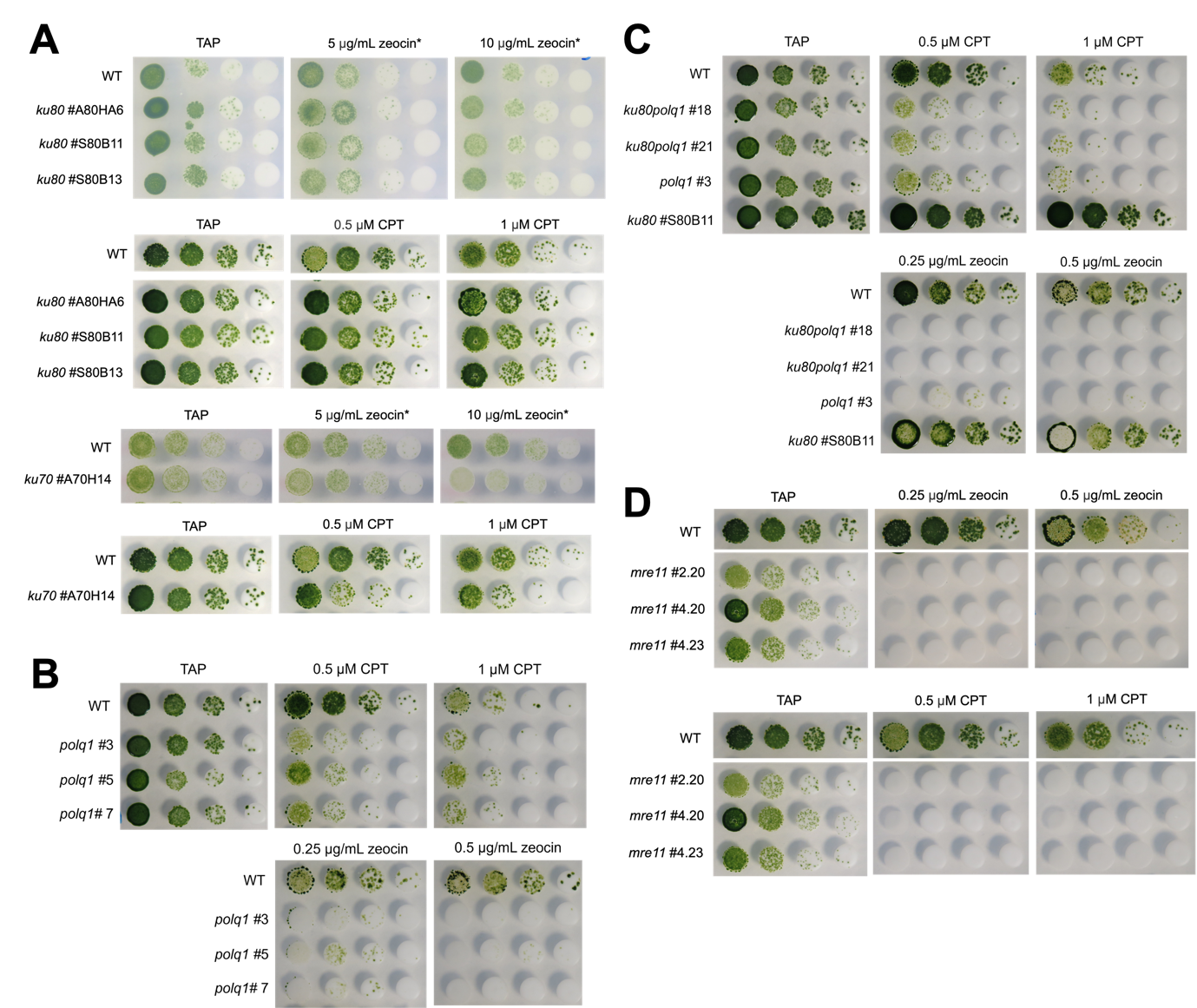


**Figure S10**. Drop assays were done using wild-type cc-1883 cells, (A) *ku70* and *ku80* lines (Ferenczi et al. 2021), (B) *polq* mutant lines (Ferenczi et al. 2021), (C) *kupolq* double mutant lines and (D) *mre11* mutant lines. This was done by normalising cells to an optical density of 750 nm (OD750) of 1 using TAP-starch and doing 10-fold dilutions on normal TAP, TAP-zeocin, and TAP-camptothecin (CPT) at the indicated concentrations.

**
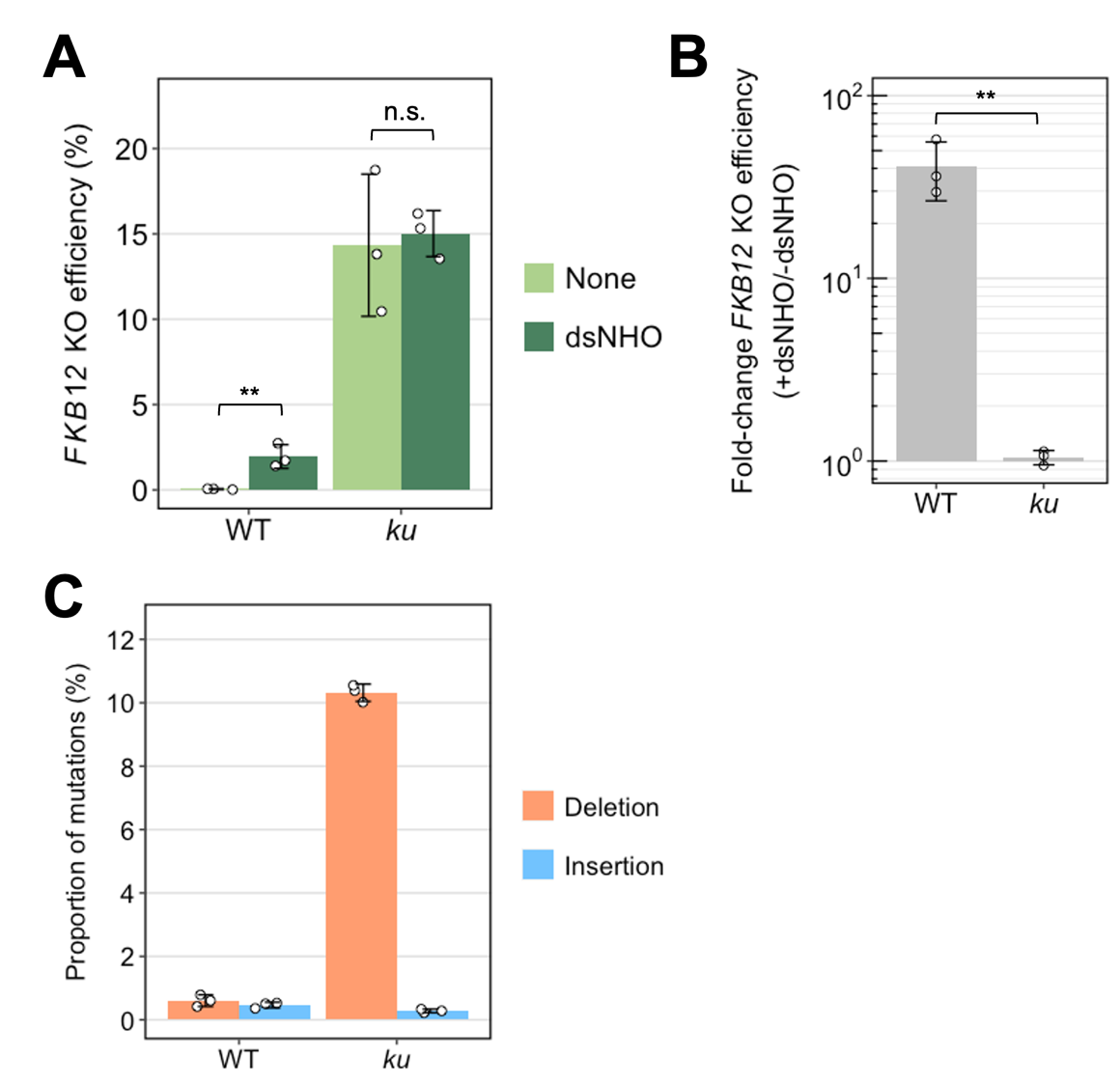
**

**Figure S11**. WT and *ku* were transformed with *FKB12* Cas12a RNP with and without 24-bp dsNHO with a different sequence (dsNHO-2, n=3). (A) *FKB12* KO efficiency and (B) fold-change *FKB12* KO efficiency between WT and *ku* was calculated. One way ANOVA test significance values are represented by asterisks. * : *p* < 0.05, ** : *p* < 0.01, *** : *p* < 0.001, n.s.: not significant. The error bars represent the standard deviation. (C) CRISPResso2 analysis (Clement et al. 2019) of amplicon data from WT and *ku* cultures treated with 24-bp dsNHO-2. The data was used to plot percentage of reads with deletion and insertions.

**
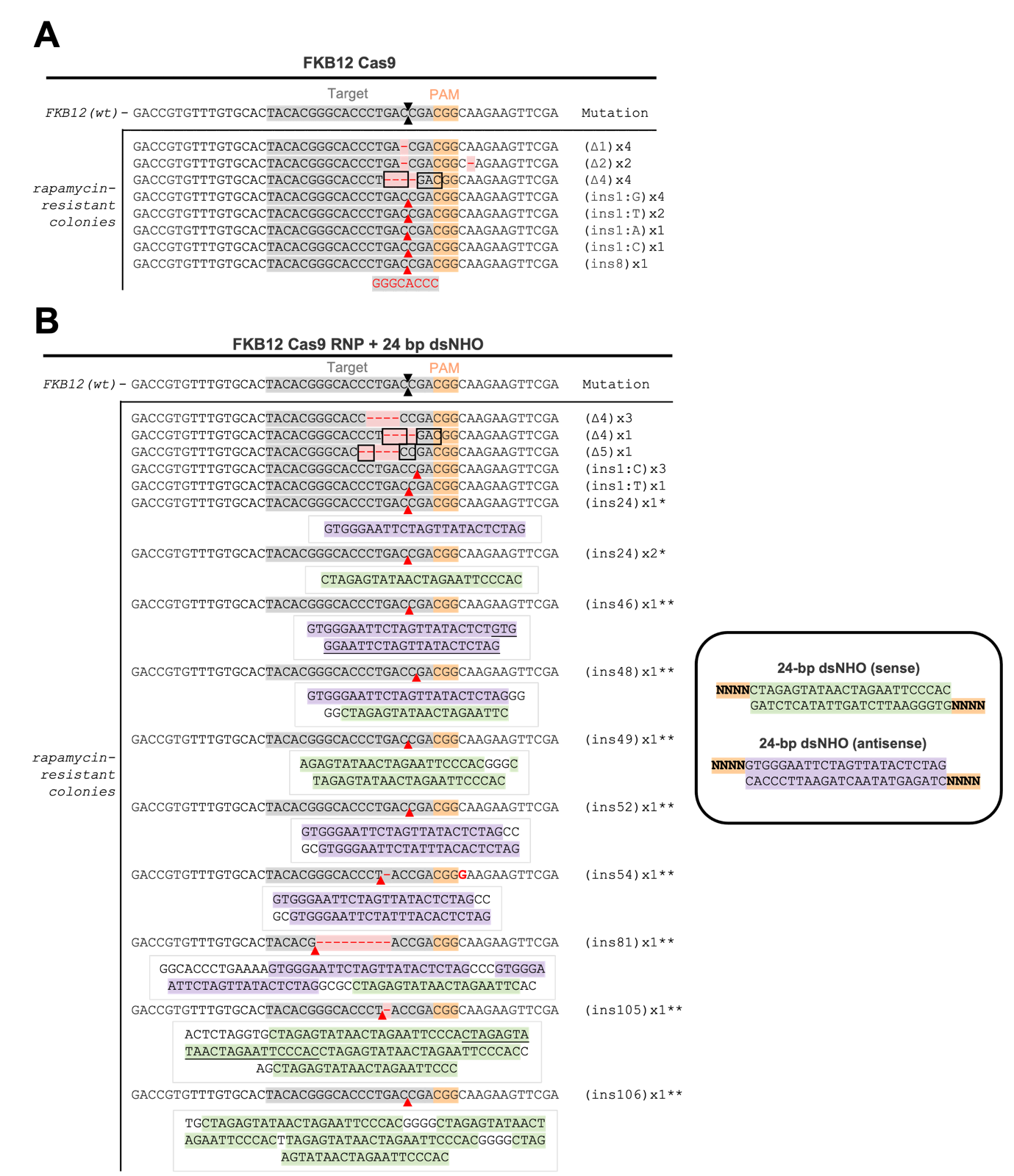
**

**Figure S12**. (A) *FKB12* locus from randomly selected rapamycin-resistant colonies treated with Cas9 RNP alone and (B) Cas9 alongside 24-bp dsNHO were amplified and sequenced with Sanger. Black triangles indicate the expected Cas9-mediated cleavage site, red triangles indicate insertion sites, and red highlights indicates sequence deviation from the WT sequence (top). Black boxes indicate potential microhomology-mediated deletions. Deletion (Δ) and insertion size (base pairs) are shown to the right of the sequence alongside the count of sequence observations. 24-bp dsNHO is seen to be inserted in two different orientations indicated in the black box. The identity of insertion sequences is shown in light grey boxes. Single and concatenated dsNHO insertions are indicated by asterisks (* and ** respectively).


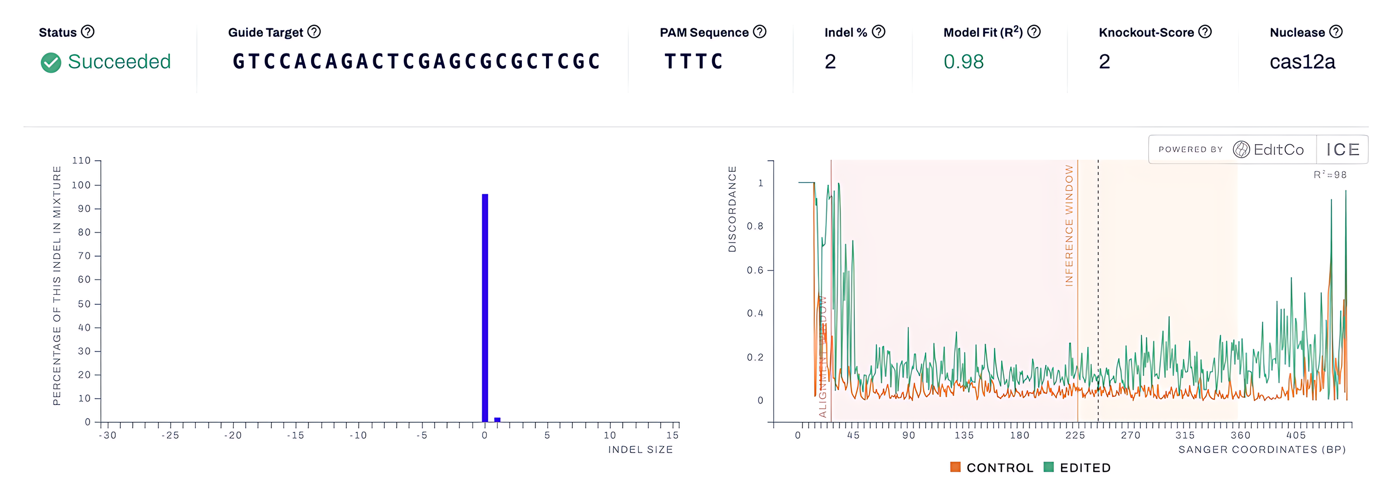


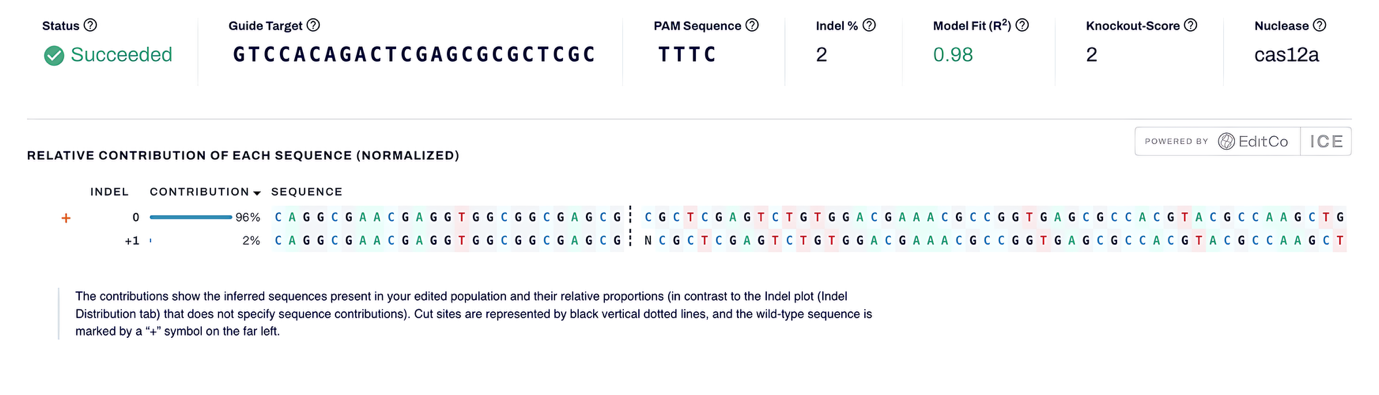


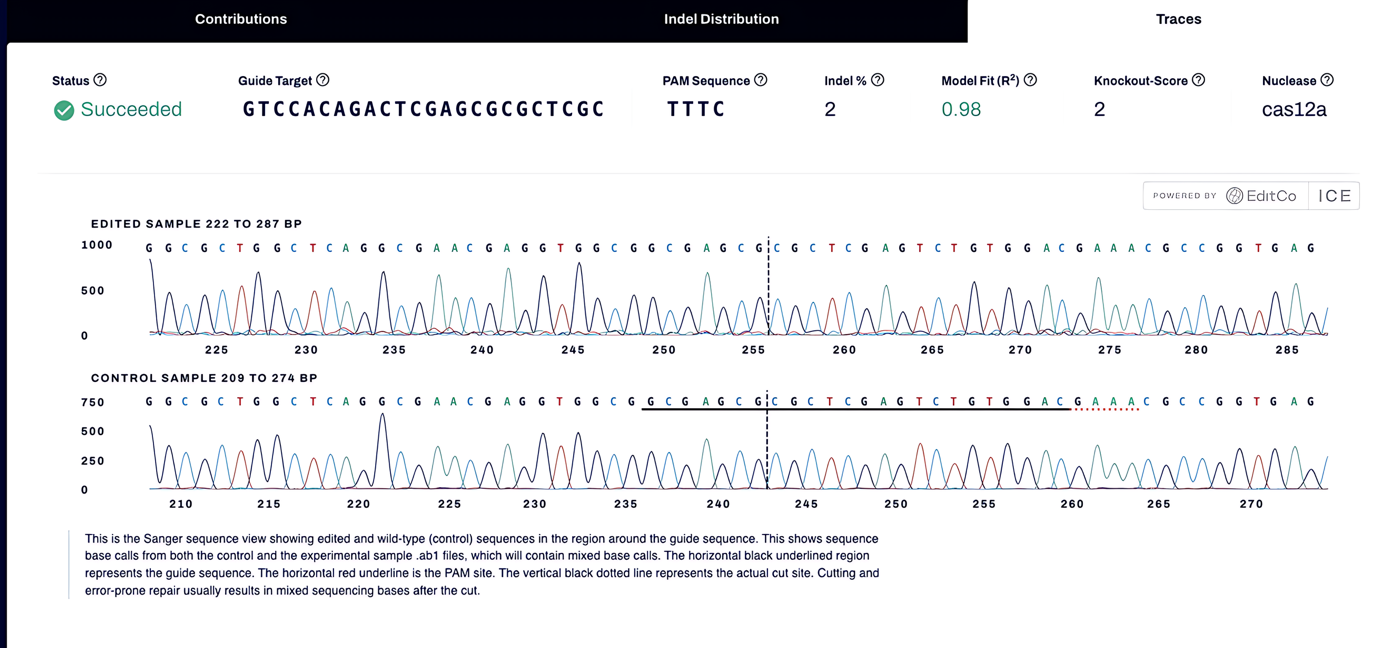


**Figure S13.** Analysis of gene editing frequency in a population of cells transfected with *PHT4-7*-specific Cas12a RNP only.

**Figure S14.** Analysis of the genotype and phenotype of *pht4-7* mutants. (A) Sequence alignment of the CrPHT4-7 mutants D#2, D#4, and D#8, generated by co-delivering Cas12a RNP and dsNHO into CC-1883 cells. D#8 harboured an 81 nt in-frame deletion and was therefore excluded from subsequent analyses. (B) Culture growth of *pht4-7* mutants and the CC-1883 wild type in TAP medium under continuous illumination at 60 µmol photons m⁻² s⁻¹ (left panel) and 350 µmol photons m⁻² s⁻¹ (right panel). Liquid cultures were incubated at 23 °C, bubbled with air for 72 h in a Multi-Cultivator photobioreactor. The initial Chlorophyl (Chl) content was set to 0.5 µg Chl(a + b)/mL. Mutants D#2 and D#4 were generated in this study using dsNHO, whereas pht4-7#7 and pht4-7#9 were produced by CRISPR–Cas12a-mediated SSTR (Ferenczi et al. 2017). (C) Chl(a + b) content after 72 h of growth at 60 and 350 µmol photons m⁻² s⁻¹ in the photobioreactor. (D) Total cellular ascorbate (Asc) content. All cultures were grown in the photobioreactor at 60 and 350 µmol photons m⁻² s⁻¹ for 72 h. Averages and standard errors are based on 3–5 independent experiments with 1-2 biological replicates each. Differences between means were assessed by ANOVA followed by Tukey’s post hoc test; means labelled with different letters are significantly different (p < 0.05).

**
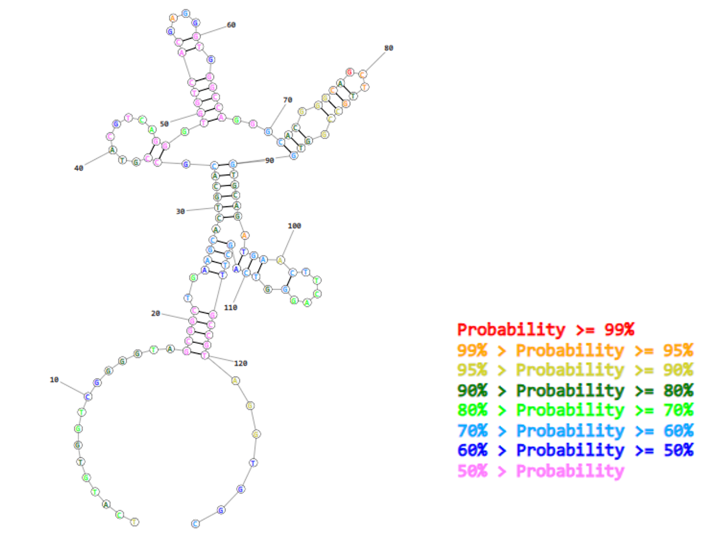
**

**Figure S15.** Predicted secondary structure the 127-nt long ssNHO (Richardson et al. 2016) using RNAstructure software (Reuter and Mathews, 2010).

**References**

- Dynan, W., and S. Yoo. 2024. “Oligomers That Bind to KU Protein—Medical College of Georgia Research Institute, Inc.” Accessed 27 January 2024. [https://www.freepatentsonline.com/6441158.html](https://eur02.safelinks.protection.outlook.com/?url=https%3A%2F%2Fwww.freepatentsonline.com%2F6441158.html&data=05%7C02%7C%7Cd11c91a7837449e952a808de5457da0b%7C2e9f06b016694589878910a06934dc61%7C0%7C0%7C639040933463989480%7CUnknown%7CTWFpbGZsb3d8eyJFbXB0eU1hcGkiOnRydWUsIlYiOiIwLjAuMDAwMCIsIlAiOiJXaW4zMiIsIkFOIjoiTWFpbCIsIldUIjoyfQ%3D%3D%7C0%7C%7C%7C&sdata=ZkGMa5SFarDUAO4SMIrNQ2PSR6NrufxVdeg5ftcZPgw%3D&reserved=0).
- Ferenczi, A., D.E. Pyott, A. Xipnitou, and A. Molnar. 2017. “Efficient targeted DNA editing and replacement in *Chlamydomonas reinhardtii* using Cpf1 ribonucleoproteins and single-stranded DNA.” *Proceedings of the National Academy of Sciences* 114, no. 51: 13567-13572.
- Ferenczi, A., Y.P. Chew, E. Kroll, C von Koppenfels, A. Hudson, and A. Molnar. 2021. “Mechanistic and genetic basis of single-strand templated repair at Cas12a-induced DNA breaks in *Chlamydomonas reinhardtii*.” *Nature Communications* 12, no. 1: 6751.
- Kim, H. K., S. Min, M. Song, et al. 2018. “Deep Learning Improves Prediction of CRISPR–Cpf1 Guide RNA Activity.” *Nature Biotechnology* 36, no. 3: 239–241.
- Moreno‐Mateos, M. A., C. E. Vejnar, J. D. Beaudoin, et al. 2015. “CRISPRscan: Designing Highly Efficient sgRNAs for CRISPR‐Cas9 Targeting In Vivo.” *Nature Methods* 12, no. 10: 982–988.
- Reuter, J. S., and D. H. Mathews. 2010. “RNAstructure: Software for RNA Secondary Structure Prediction and Analysis.” *BMC Bioinformatics* 11: 1–9.
- Richardson, C.D., G. J. Ray, N. L. Bray, and J. E. Corn. 2016. “Non-homologous DNA increases gene disruption efficiency by altering DNA repair outcomes.” *Nature Communications* 7, no 1: 12463.
